# Phenotypic and Genetic Characterization of *Aeromonas hydrophila* Phage AhMtk13a and Evaluation of Its Therapeutic Potential on Simulated *Aeromonas* Infection in *Danio rerio*

**DOI:** 10.3390/v14020412

**Published:** 2022-02-17

**Authors:** Nino Janelidze, Ekaterine Jaiani, Elene Didebulidze, Ia Kusradze, Adam Kotorashvili, Kristine Chalidze, Ketevan Porchkhidze, Tinatin Khukhunashvili, George Tsertsvadze, Dodo Jgenti, Tamaz Bajashvili, Marina Tediashvili

**Affiliations:** 1 George Eliava Institute of Bacteriophages, Microbiology and Virology, 0160 Tbilisi, Georgia; e.jaiani@pha.ge (E.J.); e.didebulidze@pha.ge (E.D.); iakusradze@pha.ge (I.K.); kristine.chalidze@gmail.com (K.C.); porchkidze.qeti@gmail.com (K.P.); tiniko960@yahoo.com (T.K.); giatserts@yahoo.com (G.T.); 2 School of Science and Technology, University of Georgia, 0171 Tbilisi, Georgia; 3 Richard Lugar Center for Public Health Research, National Center for Disease Control and Public Health, 0198 Tbilisi, Georgia; a.kotorashvili@ncdc.ge; 4 Black Sea Flora and Fauna Educational Scientific-Research Center, 6010 Batumi, Georgia; dodo.jgenti99@gmail.com (D.J.); tamazi37@mail.ru (T.B.)

**Keywords:** bacteriophage, phage genome, phage therapy, *Aeromonas hydrophila*, aquaculture, zebrafish

## Abstract

Phage therapy can be an effective alternative to standard antimicrobial chemotherapy for control of *Aeromonas hydrophila* infections in aquaculture. *Aeromonas hydrophila*-specific phages AhMtk13a and AhMtk13b were studied for basic biological properties and genome characteristics. Phage AhMtk13a (Myovirus, 163,879 bp genome, 41.21% CG content) was selected based on broad lytic spectrum and physiologic parameters indicating its lytic nature. The therapeutic potential of phage AhMtk13a was evaluated in experimental studies in zebrafish challenged with *A. hydrophila* GW3-10 via intraperitoneal injection and passive immersion in aquaria water. In experimental series 1 with single introduction of AhMtk13a phage to aquaria water at phage–bacteria ratio 10:1, cumulative mortality 44% and 62% was registered in fish exposed to phage immediately and in 4 h after bacterial challenge, correspondingly, compared to 78% mortality in the group with no added phage. In experimental series 2 with triple application of AhMtk13a phage at ratio 100:1, the mortality comprised 15% in phage-treated group compared to the 55% in the control group. *Aeromonas hydrophila* GW3-10 was not detectable in aquaria water from day 9 but still present in fish at low concentration. AhMtk13a phage was maintained in fish and water throughout the experiment at the higher concentration in infected fish.

## 1. Introduction

The aquaculture industry has been expanding significantly worldwide in the past decades. In 2018, total fish production, trade, and consumption reached an all-time record equaling 114.5 million tons in live weight [1]. Aquaculture is one of the fastest growing and developing sectors in South Caucasus region, including Georgia. Rainbow trout is the most common cultured fish species in Georgia, comprising 61% of the total fish production [2].

One of the main challenges for the development of aquaculture worldwide and also in Georgia are bacterial infections that may lead to massive death in fish and, correspondingly, to large financial losses. Efforts to keep farmed fish free of disease are important for both fish welfare and fish farmers. The problem related to occurrence and spread of infections is especially acute for fish hatcheries and larvae producing facilities, and also juvenile fish ponds, where mortality rates usually are significantly higher compared to adult fish. The freshwater fish bacteriosis in the natural environment and in fish farms is mainly caused by a number of *Aeromonas* species [3]. *Aeromonas hydrophila*, a Gram-negative, rod-shaped facultatively anaerobic bacterium, is a natural inhabitant of fresh and brackish waters. *Aeromonas hydrophila* frequently causes disease outbreaks in wild and cultured fish leading to *Aeromonas* septicemia and ulcerative infections [4]. Recent studies have reported isolation of multidrug resistant (MDR) *A. hydrophila* from diseased fish and aquatic systems worldwide [5,6]. 

Various antimicrobials—chemicals, dyes, and antibiotics—have been used in aquaculture for treatment of sick fish and for prevention of bacterial infections. Use of antibiotics to control fish bacteriosis, including *Aeromonas* infections, is still the most widely used approach that guarantees reduction in morbidity and mortality, and contributes to significant advances in the health of the population. The public health hazards related to use of antimicrobials in aquaculture include development and spread of antimicrobial-resistant (AMR) bacteria and resistance genes, along with the presence of antimicrobial residues in aquaculture products and the environment [7]. The rising drug resistance among aquatic bacteria and adverse effects of antibiotics lead to a common understanding that use of antibiotics for prevention of fish diseases should remain low and should not be a primary treatment option in fish farming practices [8]. The use of antibiotics in aquaculture is under strict control in Europe and fish farmers are advised to use other complementary control strategies [9,10,11]. To follow the principle “prevention is better than treatment”, different types of vaccines, predominantly killed and live attenuated vaccines have been used for prevention of bacterial infections in farmed fish. Application of probiotics and prebiotics is a considerably new approach [12,13]. Prophylactic treatments are mostly confined to the hatchery, the juvenile or larval stages of aquatic animal production, and are thought to be more common in small-scale production units [13].

Biological control of diseases, including phage therapy and prophylaxis, is currently considered as a best approach for aquaculture [14,15]. Application of therapeutic phages in aquatic animals is promising due to several reasons: (i) bacteriophages are viruses that infect only bacteria and are highly specific (mainly species, or even strain specific); (ii) phage reproduction inside the organism (human, animals, etc.) takes place only when host bacteria are present, so they do not accumulate in the organism and in the environment; (iii) phages have ability for fast propagation and high burst size that leads to lysis of bacterial host; (iv) phages are immunomodulators and can promote specific mechanisms of bacterial clearance; (v) high natural abundance of phages in aquatic environment (ranging from 10^4^ to 10^8^ mL^−1^) makes the phage therapy in fish more tolerable than other approaches; (vi) regulations in aquaculture concerning use of biopreparations are considerably milder than in medicine that simplifies the implementation of phage-based treatments and products in aquaculture. Perhaps one of the earliest phage therapy applications in aquaculture was described in 1980s by Wu et al., when the strongly lytic phage AH1 was used to treat *A. hydrophila*-infected loaches [16]. Numerous in vitro assays and in vivo studies evaluated the potential of bacteriophages (individual phages and phage mixtures) and phage lytic enzymes to effectively combat fish pathogenic bacteria including multidrug resistant *A. hydrophila* [17,18,19,20,21,22].

In the last decade there has been a growing interest in Europe toward use of biological preparations, particularly phages, in aquaculture. The same trend in Georgia can be evidenced by the increased inquiry from aquaculture companies to the G. Eliava Institute of Bacteriophages, regarding development of aquaculture-targeted phage products. Most frequently this applies to fish bacterial infections, caused by *Aeromonas* spp. Currently, no commercial phage preparation is produced for aquaculture needs. Well-designed experimental studies with customized phage preparations are of high demand in order to demonstrate effectiveness and reproducibility of phage-based treatment. 

This study aimed at the detailed characterization of two new *A. hydrophila* specific phages and evaluation of their potential for infection control in aquaculture. In order to assess the antibacterial efficacy of lytic phage AhMtk13a to combat *A. hydrophila* infection and to reduce the fish mortality, the experimental studies were conducted on the model of zebrafish challenged with *A. hydrophila* GW3-10, a virulent isolate from the sick trout. 

## 2. Results 

### 2.1. Isolation and Characterization of A. hydrophila Specific Bacteriophages

#### 2.1.1. Isolation of *A. hydrophila* Specific Bacteriophages

For the isolation of *A. hydrophila*-specific phages, water samples from the Mtkvari River (in the suburbs of Tbilisi, Georgia) were enriched with *A. hydrophila* GW3-10. The number of phage particles in the primary phage lysate was amplified on the host strain by use of soft agar overlay method [23]. After propagation of the primary phage lysate (that often can be a natural mixture of phages) on the *A. hydrophila* GW3-10, two morphologically different phage plaques were observed on the bacterial lawn: (i) predominating larger size (2.5–3 mm diameter) negative colonies with clear centers surrounded by narrow halozon; (ii) the smaller, turbid plaques with diameter 1–1.5 mm. For each of these two *Aeromonas* phages with different plaques, the pure lines were obtained using five cycles of purification to ensure a single phage-strain population. The phage forming larger negative colonies was given the name vB_AhMtk13a and the phage with small size plaques was named as vB_AhMtk13b.

#### 2.1.2. Phage Virion Morphology

**Figure 1:**
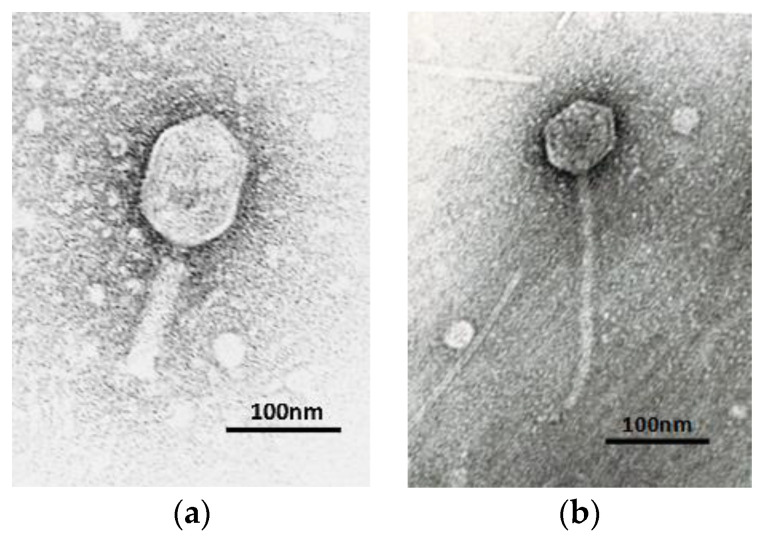
Virion morphology of *A. hydrophila*-specific bacteriophages: (**a**) phage AhMtk13a; (**b**) phage AhMtk13b.

The two *Aeromonas* phages after propagation to high titers were examined for phage virion morphology by transmission electron microscopy (TEM). The study showed that both phages by morphological characteristics belong to the order *Caudovirales* (Figure 1a,b). The phage AhMtk13a has the elongated icosahedral head (118 ± 3 nm × 86 ± 3 nm) and contractile tail (123 ± 3 nm × 23 ± 4 nm) and attributed to the *Myoviridae* morphotype. AhMtk13b phage has symmetrical icosahedral head (68 ± 2 nm × 68 ± 2 nm) and long noncontractile tail (227 ± 4 nm × 11 ± 2 nm), and belongs to the *Siphoviridae* morphotype.

#### 2.1.3. Whole Genome Sequencing of AhMtk13a and AhMtk13b Phages and In Silico Analysis

**Figure 2:**
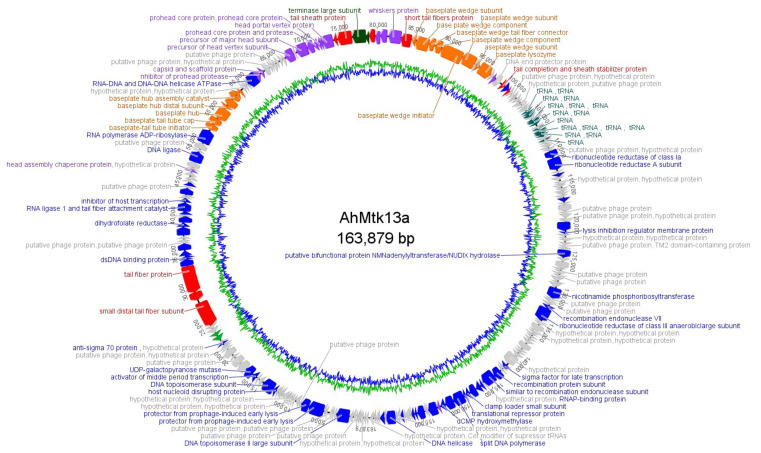
Phage AhMtk13a genome map.

**Figure 3:**
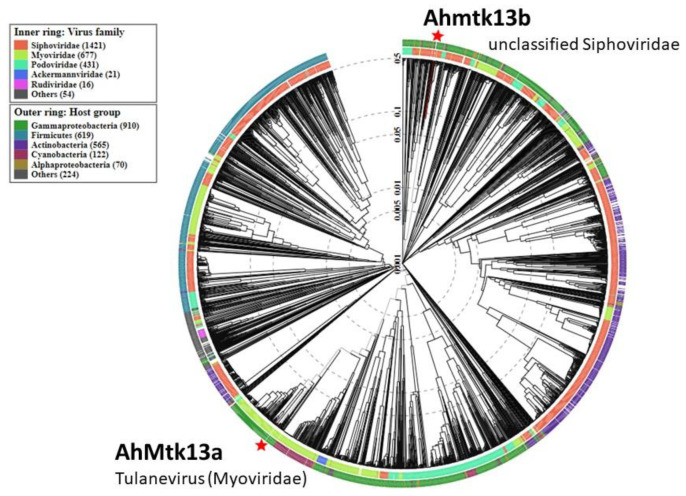
Phylogenetic tree based on protein level constructed by VIP tree [24].

Phage AhMtk13a contains dsDNA with size 163879 bp in length, C+G content is 41.21%. Analysis of sequencing data identified 151779 bp as gene coding region and total 246 ORFs were predicted. For most of ORFs start codons were ATG (M); however, some ORFs start with GTG (V) and TTG (L). Among 246 ORFs, 131 were annotated with an assigned function. Genes with predicted functions were grouped as follows: packaging module (2 ORFs), capsid morphogenesis module (15 ORFs), tail morphogenesis module (9 ORFs), baseplate proteins (16 ORFs), host cell lysis module (2 ORFs), and DNA metabolism and replication module (56 ORFs). Phage genome does not contain lysogeny control genes. Sixteen tRNAs were identified (Figure 2 and Figure 3). Phage AhMtk13a is a member of family *Myoviridae*; genus, Tulanevirus. 

**Figure 4:**
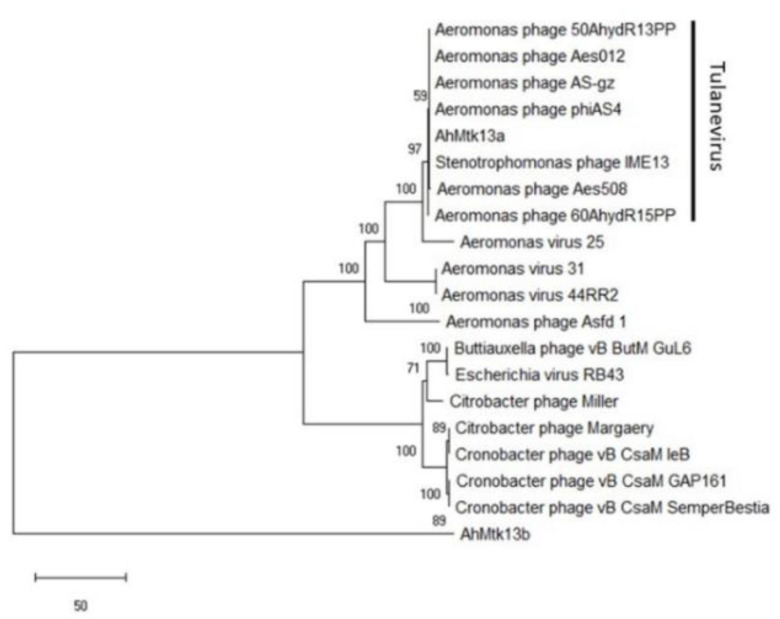
Phylogenetic trees of terminase large subunit, constructed with AhMtk13b, AhMtk13a and reference phages identified by BLASTP hits (e-value 0.0, identity >70%).

BLASTn similarity search revealed that eight phages were similar to AhMtk13a (coverage 97–92%, identity 97–95%, respectively, e-value 0.0). However, among these phages average nucleotide identity is significantly higher (ANI −95–97%) for six phages, that indicates that AhMtk13a and all abovementioned six *Aeromonas* phages (50AhdR13PP, Aes012, AS-gz, phiAS4, Aes508, 60AhydR15PP) and *Stenotrophomonas* phage IME13 (Figure 4) have similar genome organization. The large terminase subunit of AhMtk13a phage and the most closely related phage proteins (large terminase subunit) (e-value 0.0, identity >70%) were used to construct a phylogenetic tree (Figure 4).

**Figure 5:**
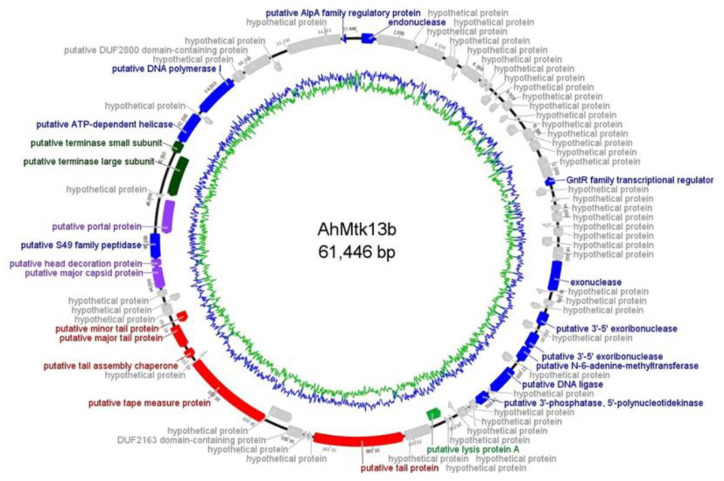
Genome map of AhMtk13b.

Phage AhMtk13b has been identified as unclassified *Siphoviridae* (Figure 3) with dsDNA of 61446bp in length and CG content of 62.01%. The gene coding percentage is 94.2% and a total of 75 ORFs were identified, with start codons ATG (M); GTG (V) and TTG (L). Twenty-three ORFs from 75 were annotated functionally, which include gene encoded structural proteins as follows: major tail protein, tape measure protein, tail protein, major capsid protein, head decoration protein, tail assembly chaperon, portal protein; proteins involved in packaging as terminase large subunit and terminase small subunit. The products of the 10 ORFs belonged to the DNA metabolism replication modules. Only 1 ORF was identified as gene responsible for host lysis. Phage genome does not contain lysogeny control genes. No tRNAs were identified (Figure 5).

**Figure 6:**
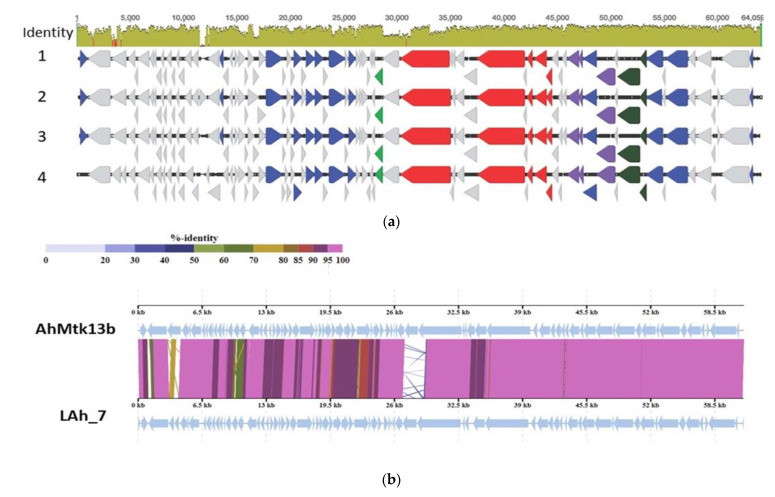
Genome comparison: (**a**) Comparative genome maps of AhMtk13b (1) and three *Aeromonas* phages ((2) BUCT551 (GeneBank: MT952005.1); (3) vB_AhyS-A18P4 (GeneBank: MN317029.1); (4) LAh_7 (GeneBank: MK838113.1)) ORFs are colored according to functions of the gene products: green—packaging module; purple—capsid morphogenesis module, red—tail morphogenesis module; light green—lysis module; blue—DNA metabolism and replication; (**b**) genome alignments of AhMtk13b and LAh_7 (GeneBank: MK838113.1).

Similarity search revealed homology phage of AhMtk13b with three *Aeromonas* phages available on CBI database. AhMtk13b is highly similar only to *Aeromonas* phage LAh_7 (MK838113.1) with average nucleotide identity (ANI) 95.94%, which indicates that these two phages are same species (Figure 6b) [25]. However, all four phages—AhMtk13b and previously sequenced three phages BUCT551, AhyS-A18P4, and LAh_7 from the NCBI database—display similar morphological features (Figure 6a). 

#### 2.1.4. Phage Host Range

The host range of AhMtk13a and AhMtk13b phages was tested on 49 *Aeromonas* strains, including 46 Georgian isolates and 3 reference strains of *A. hydrophila* and *A. salmonicida* (Table S1). 

The phage AhMtk13a expressed the lytic activity against the majority of tested *A. hydrophila* strains (29 out of 40), including reference strain *A. hydrophila* CIP103770. The AhMtk13b phage revealed similar but much narrower host range lysing, only 13 out of 40 *A. hydrophila* strains. The strains of other *Aeromonas* species appeared to be resistant to both phages, thus expressing characteristics of species-specific (to *A. hydrophila*) phages. Since AhMtk13a phage showed broader host-range (73%), also covering the strains lysed by phage AhMtk13b, it was selected for further studies as a candidate therapeutic phage (Table 1 and Table S2).

#### 2.1.5. The Phage One-Step Growth Cycle

**Figure 7:**
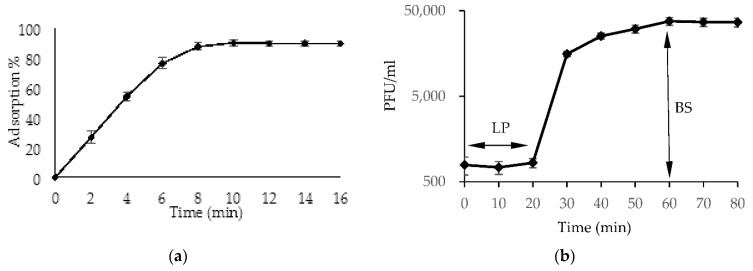
(**a**) Adsorption curve of AhMtk13a phage. (**b**) Single-step growth curve of AhMtk13a phage. The results are the mean values of three independent tests. Standard deviations (SD) are indicated.

One-step growth experiments with the phage AhMtk13a were performed on the host strain *A. hydrophila* GW3-10, in order to determine phage adsorption, latent period, and burst size. Based on results obtained, the phage AhMtk13a achieved a maximal adsorption of 90% within 10 min at the temperature 30 °C (Figure 7a). The latent period of phage AhMtk13a comprised 20 min and the average burst size, 160 phage particles per infected cell (Figure 7b). These results were suggestive for the virulent nature of AhMtk13a phage.

#### 2.1.6. Lysis Stability of AhMtk13a in Liquid Culture 

The lysis stability of AhMtk13a phage in TSB was studied on the host strain *A. hydrophila* GW3-10 according to the method of Appelmans [26] (Table 2). At all phage–bacteria ratios, the lysis was registered during first 6 h of incubation. After 24 h the visible bacterial growth (VBG), still less than in control tubes, was detected in reaction tubes with phage–bacteria ratio 0.01 and 0.001. After 48 h of incubation, AhMtk13a phage maintained the lysis at the MOI 1000, 100, 10, and 1. The VBG was detected in reaction tubes at MOI 0.1 with the turbidity matching to 0.5 by McFarland turbidity standard (MFTS). The intensity of bacterial growth in reaction tubes with MOI 0.01 and 0.001 was increased in 48 h compared to 24 h, but still with the lower turbidity compared to the control tubes, where turbidity matched MFTS 3 (Table 2).

### 2.2. Stability and Infectivity of Bacteriophage AhMTK13a in Different Environmental Conditions

#### 2.2.1. Survival in Different Liquid Environments

**Figure 8:**
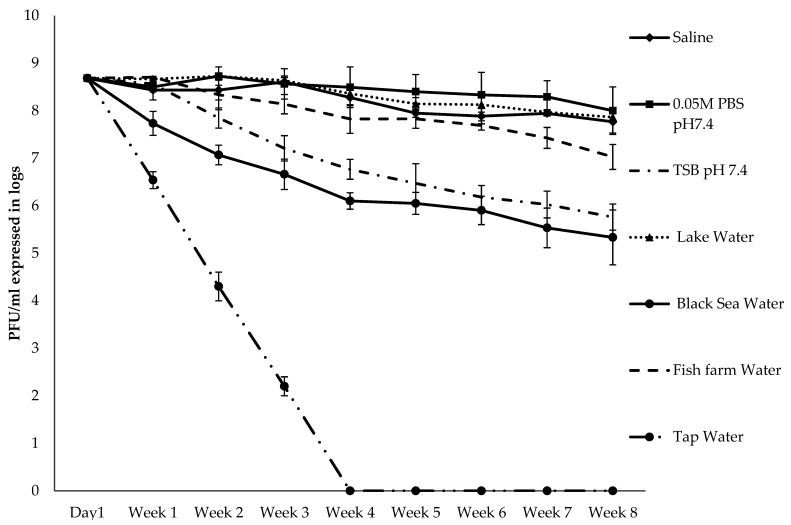
The survival of AhMtk13a phage in different liquid environments: saline, PBS, TSB, lake water, Black Sea water, fish farm water, and tap water. The results are the averages of three parallel experiments with geometric SD shown as the vertical lines.

Stability of bacteriophage AhMtk13a was tested by measuring the infectivity of phage particles exposed to various liquid environments (saline, PBS, TSB, Lake Lisi water, Black Sea water, fish farm water, tap water) during eight weeks. The phage AhMtk13a expressed the highest stability in PBS, saline, and brackish water of Lisi Lake (≤1 log decrease in viable phage particles during eight weeks) compared to the TSB where the number of infective phage particles was decreased by 3 logs (Figure 8). During the same period, a slight drop (1.7 log) in the phage counts was registered in the water from the fish farm (river water with salinity 0.2 ppt). The AhMtk13a phage revealed less stability in the Black Sea water (salinity 17 ppt) where the number of infective phage particles was decreased by 3.4 log. AhMtk13a phage demonstrated inability to survive in tap water: the sharp decrease in phage counts started from the first week and in three weeks the number of viable phage particles was below the detection limit.

#### 2.2.2. Influence of Temperature on AhMtk13a Phage Survival

**Figure 9:**
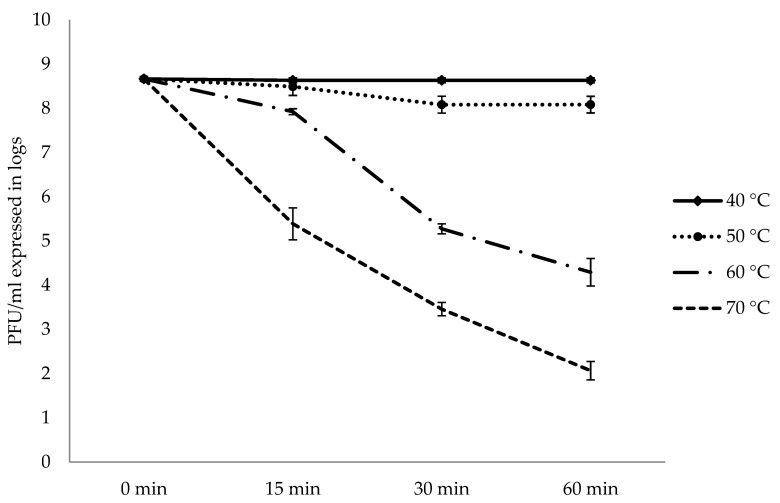
The survival of AhMtk13a phage at different temperatures. The results are the averages of three parallel experiments with geometric SD shown as the vertical lines.

There was no reduction in AhMtk13a phage infectivity after incubation in TSB during 1 h at 40 °C and the only slight decrease—by 0.6 logs (compared to the starting point)—was registered at 50 °C. However, after 1 h exposure of AhMtk13a phage to the elevated temperatures, 60 and 70 °C, the number of infective particles decreased significantly (*p* < 0.05) by 4.4 and 6.6 logs, correspondingly (Figure 9). 

#### 2.2.3. Influence of Acidic and Alkaline Environment on Survival of AhMtk13a Phage

**Figure 10:**
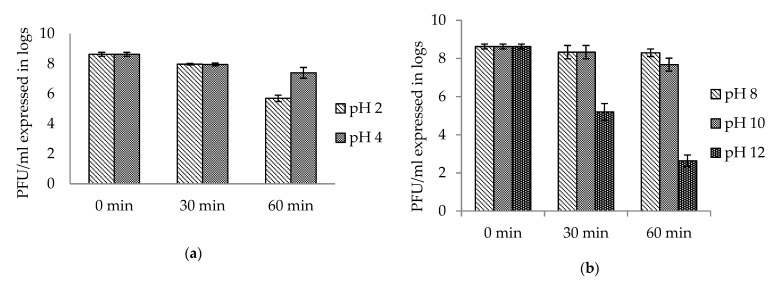
(**a**) Survival of AhMtk13a phage in acidic environment. (**b**) The survival of AhMtk13a phage in alkaline environment. The results are the averages of three parallel experiments with geometric SD shown as the vertical lines.

AhMtk13a phage was tested for stability under acidic and alkaline conditions at 30 °C. It showed high survival at pH 4, with only 1 log decrease in phage counts after 1 h, while at pH 2, the decrease in infective phage particles comprised 3 logs (Figure 10a). The viable phage counts at pH 8 remained unchanged and decreased by up to 1 log at pH 10 in 1 h. At pH 12, the decrease in phage viability by 3.4 log was already registered in 30 min with a more sharp decrease (by 6 logs) in 1 h (Figure 10b).

### 2.3. A. hydrophila GW3-10 Pathogenicity Experiments: Development of Infection Model 

Estimation of Zebrafish Mortality Depending on the Concentration of *A. hydrophila* GW3-10.

It is well known that zebrafish represent a perfect model for experimental studies on evaluation of efficacy and safety of different antimicrobial substances including phages [20,27,28,29,30].

The aim of our experiments was to determine the disease-causing capacity of *A. hydrophila* GW3-10, isolated from diseased trout serving as the host strain for the phage AhMtk13a. For this purpose, the adult zebrafish was infected with *A. hydrophila* GW3-10 using different routes of administration and observed during 14 days. At the initial stage, to simulate natural route of infection in the model system, we used static immersion of fish to aquaria water containing two different bacterial concentrations ×10^5^ and ×10^7^ CFU/mL. However, fish mortality under these conditions was not observed.

To increase the disease severity and mortality in the model fish, experimental protocol was modified through (i) infliction of the minor injuries/dermal abrasions to fish or (ii) intraperitoneal injections of model fish, both routes followed by immersion of the fish in aquaria water containing ×10^7^ CFU/mL of *A. hydrophila* GW3-10. No fish mortality was observed in the scratched fish exposed to aquaria water without bacterial insemination (control group). For the fish with dermal abrasion exposed to aquaria water containing pathogen, a low-level mortality (17%) was achieved on days 2–3.

**Figure 11:**
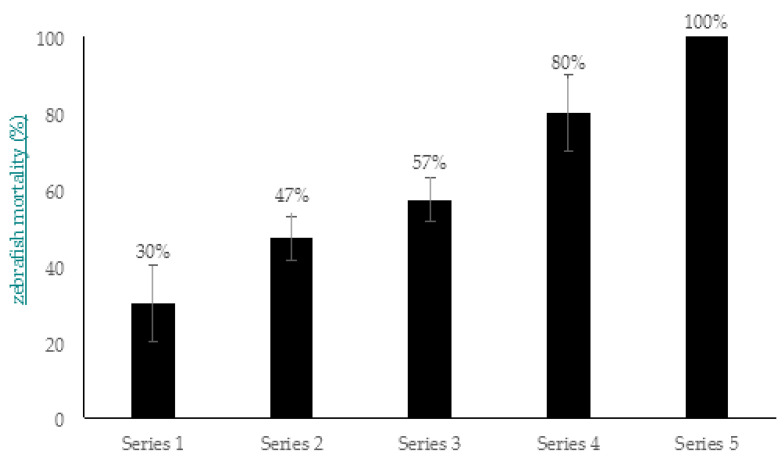
Mortality in zebrafish caused by different concentrations of *A. hydrophila* GW3-10 inoculated by intraperitoneal injection (0.1 mL) and exposed to the aquaria water containing ×10^7^ CFU/mL *A. hydrophila* GW3-10. Series 1—fish engrafted with ×10^3^ CFU/mL *A. hydrophila* GW3-10; Series 2—fish engrafted with ×10^5^ CFU/mL *A. hydrophila* GW3-10; Series 3—fish engrafted with ×10^6^ CFU/mL *A. hydrophila* GW3-10; Series 4—fish engrafted with ×10^7^ CFU/mL *A. hydrophila* GW3-10; Series 5—fish engrafted with ×10^8^ CFU/mL *A. hydrophila* GW3-10. The results are the averages of three parallel experiments with SD shown as the vertical lines.

The higher mortality rates were achieved in the experiments where the intraperitoneal injection of zebrafish (with different bacterial concentrations) was used in combination with static immersion to aquaria water (×10^7^ CFU/mL *A. hydrophila* GW3-10). It was shown that zebrafish mortality was increasing in a dose-dependent manner: for zebrafish infected with ×10^3^ CFU/mL, 30% mortality was observed, and 47% and 57% mortality was observed for ×10^5^ and ×10^6^ CFU/mL, respectively (Figure 11). The highest mortality rates (80% and 100%) were registered in zebrafish ingrafted with ×10^7^ and ×10^8^ CFU/mL of *A. hydrophila* GW3-10. It should be mentioned the registered death in fish occurred during the first 4 days for injected bacterial concentrations from ×10^3^ to ×10^7^ CFU/mL, while the injected dose ×10^8^ CFU/mL led to occurrence of all fish death within 24 h.

Experimental strain was reisolated from all dead fish samples and was confirmed to be *A. hydrophila* GW3-10 based on cultural characteristics and the high susceptibility to bacteriophage AhMtk13a. The number of bacteria isolated from homogenized fish samples varied from ×10^6^ to ×10^8^ CFU/fish. 

The results of the pathogenicity experiments showed capability of target pathogen *A. hydrophila* GW3-10 to establish artificial infection in zebrafish with the high mortality rates under developed conditions. These conditions were further used for evaluation of phage-based antimicrobial treatment in experimental studies in the zebrafish model. 

### 2.4. Estimation of Antibacterial Efficacy of AhMtk13A Phage in the Experimental A. hydrophila GW3-10 Infection in Zebrafish Model: Experimental Series 1

Experimental series 1 aimed at the study of antibacterial efficacy of AhMtk13a phage in vivo. *A. hydrophila* infection model in zebrafish was used based on static immersion (aquaria water containing ×10^7^ CFU/mL) combined with the intraperitoneal injection of zebrafish with ×10^7^ CFU/mL *A. hydrophila* GW3-10. 

Prior to beginning experimental series 1, a small-scale experiment was conducted for evaluation of safety of phage preparation to be used in the challenge experiments. Forty fish in 4 aquaria (3 experimental parallels containing phage and 1 control with saline added instead of phage) were monitored for 2 weeks. No visible changes in physical and behavioral parameters such as body conformation, emaciation, swimming behavior, or signs of possible sickness (hemorrhages, ulceration, and discoloration) were observed.

In experimental series 1, the AhMtk13a phage was added directly to the aquaria water to a final concentration ×10^8^ PFU/mL. In total, 270 healthy adult zebrafish were equally divided into six groups (4 experimental and 2 control groups in 3 parallels each): group I—fish injected with *A. hydrophila* GW3-10, placed in the aquaria containing the same bacteria and immediately added AhMtk13a phage; group II—fish injected with *A. hydrophila* GW3-10, placed in the aquaria with the same bacteria and the phage added in 4 h after infection; group III—fish exposed to AhMtk13a phage containing aquaria 30 min before intraperitoneal injection with *A. hydrophila* GW3-10 and adding the same pathogen to the aquaria water; group IV (bacterial control)—fish injected with the *A. hydrophila* GW3-10 and kept in the aquaria containing the same bacterial pathogen; group V (phage control)—fish injected with saline and placed in the aquaria containing the experimental phage only; and group VI (vehicle control)—fish received saline only through intraperitoneal injection. Neither phage nor bacteria were added to these aquaria.

#### 2.4.1. Protective Effect of AhMtk13a Phage Administration on Infected Zebrafish: Comparative Mortality Rates 

**Figure 12:**
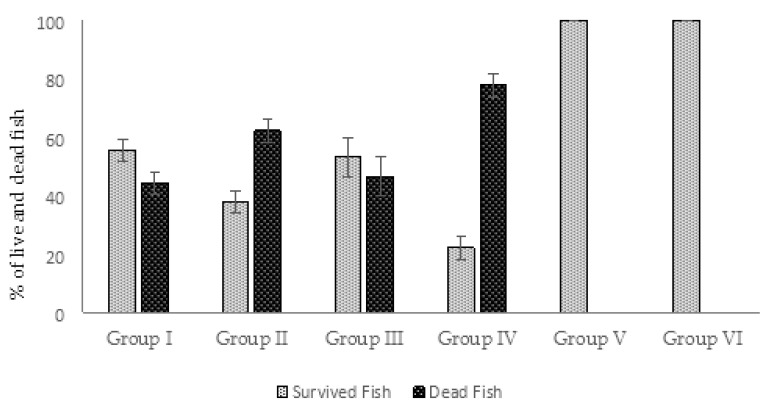
Protective effect of AhMtk13a phage on zebrafish infected with *A. hydrophila* 3–10: survival and death rates (%) in different experimental groups. Group I—fish injected with *A. hydrophila* GW3-10, placed in the aquaria with the same bacteria added and immediately treated with the phage; Group II—fish injected with *A. hydrophila* GW3-10, placed in the aquaria with the same bacteria added and treated with the phage in 4 h after infection; Group III—fish exposed to phage AhMtk13a containing aquaria 30 min before intraperitoneal injection with *A. hydrophila* GW3-10 and adding the same pathogen to the aquaria water; Group IV (bacterial control)—fish injected with the *A. hydrophila* GW3-10 and kept in the aquaria containing the same bacterial pathogen; Group V (phage control)—fish injected with saline and placed in the aquaria containing the experimental phage only; Group VI (vehicle control)—fish received saline only through intraperitoneal injection. Neither phage nor bacteria were added to these aquaria. The results are the averages of three parallel experiments with SD shown as the vertical lines.

The protective effect of AhMtk13a phage manifested as reduction of mortality in fish was monitored for 14 days after challenge of zebrafish with ×10^7^ CFU/mL of *A. hydrophila*. The death of infected zebrafish was registered within the first 4 days after bacterial challenge. The highest cumulative mortality (78%) was registered in group IV, received no phage treatment (Figure 12). Fish in this group began to already die in 4 h after the bacterial challenge and the last death was registered at day 4 in one parallel only. The phage-treated group II was on second position by 62% of cumulative mortality with the dynamics of the death registration similar to group IV (Figure 12). In the phage-treated group I (simultaneous administration of phage) and phage-treated group III (fish received phage 30 min before bacterial challenge), the first mortalities in fish were registered in 24 h and continued up to 4 days. The average cumulative mortality in these groups comprised 44% and 47%, correspondingly, being significantly (*p* < 0.05) lower compared to group IV (Figure 12). No mortality was observed in experimental groups V (phage control) and VI (vehicle control) during the entire course of experiment. 

*A. hydrophila* GW3-10 in the concentration from 1 × 10^7^ to 5 × 10^7^ CFU/fish was isolated from all tested dead fish individuals. The randomly selected bacterial colonies grown on Aeromonas selective agar were checked by phage spot test and were shown to be susceptible to AhMtk13a phage.

#### 2.4.2. Dynamic Changes in Abundance of Phages and Bacteria in Zebrafish (Experimental Series 1)

Dynamic changes in phage and bacterial numbers as a result of phage–bacteria interaction in zebrafish were studied in group I compared to control groups IV, V, and VI. 

In total, 180 healthy adult zebrafish were used in challenge experiments according to the experimental protocol described in Materials and Methods, Section 4.9. The fish from group V were monitored for the presence of AhMtk13a phage every day during 14 days. The same testing in groups I and IV was done during the first 5 days and at the last day of the experiment because of the expected reduction in fish numbers due to the mortality. 

**Figure 13:**
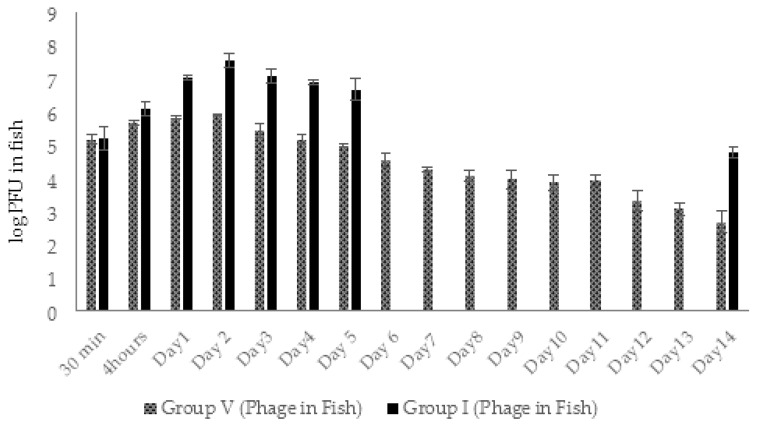
Dynamic changes in AhMtk13a phage concentration in phage-treated fish: Group I—fish injected with *A. hydrophila* GW3-10, placed in the aquaria with the same bacteria added and immediately treated with the phage; Group V (phage control)—fish injected with saline and placed in the aquaria containing the phage only. The results are the averages of three parallel experiments with geometric SD shown as the vertical lines.

In groups I (experimental) and V (phage control), the phage particles in fish bodies were detected already in 30 min after adding phage to aquaria water (final concentration ×10^8^ PFU/mL) (Figure 13). The amount of phage in examined fish from groups V and I was in range of ×10^5^ PFU/fish. In 4 h, the number of phage particles in group I fish rose ~10 times (Figure 13) and the maximal amount (3.7 × 10^7^ PFU/fish) of phage particles was detected in 48 h. This indicates active propagation of the AhMtk13a phage on bacterial pathogen. In group V the number of phages in fish increased only three times in 4 h comprising 4.5 × 10^5^ PFU/fish and reached the maximal numbers in 48 h (6.4 × 10^5^ PFU/mL), which is still 1.6 logs lower compared to the fish from group I (Figure 13).

In both groups the gradual decline in phage abundance in fish started from day 3 with more obvious decrease in group V compared to group I. Interestingly, at the end of the experiment the concentration of the phages in group I fish was 2.1 logs higher compared to group V fish (Figure 13). 

**Figure 14:**
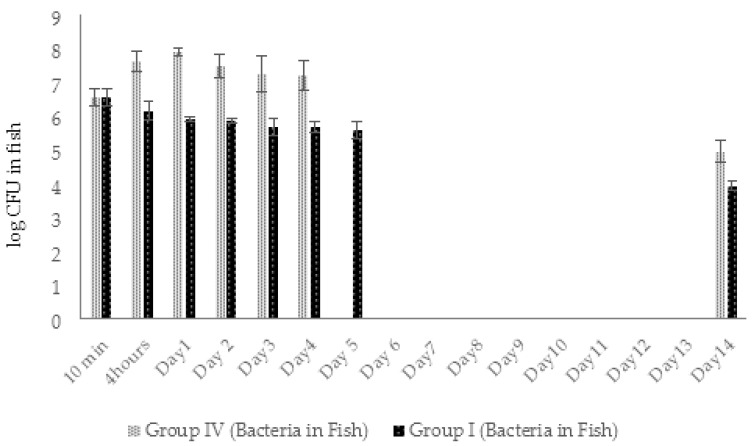
Dynamic changes in *A. hydrophila* GW3-10 concentration in fish: Group I—fish injected with *A. hydrophila* GW3-10, placed in the aquaria with the same bacteria added and immediately treated with the AhMtk13a phage; Group IV (bacterial control)—fish injected with the *A. hydrophila* GW3-10 and kept in the aquaria containing the same bacterial pathogen. The results are the averages of three parallel experiments with geometric SD shown as the vertical lines.

The average amount of *A. hydrophila* GW3-10 in fish in 10 min after injection comprised 4 × 10^6^ CFU/fish. In 4 h, pathogen concentration in infected fish of group IV increased by 1 log (Figure 14). At the same time, the first cases of the fish death were registered. The last death in group IV fish was registered at day 4. During these 4 days the bacterial concentration in fish body was in range of 2–8 × 10^7^ CFU/fish and resulted in 29% of average daily mortality. At day 14, bacterial concentration in surviving fish was in range of 1 × 10^5^ CFU/fish (Figure 14). 

In group I (phage-treated fish), bacterial concentrations decreased approximately 2.5 times in 4 h, continued to decline gradually, and comprised 4.3 × 10^5^ CFU per fish by day 4. The decline in bacterial counts positively correlated with increase in phage numbers in group I. During the first 4 days, reduction in bacterial numbers in fish of group I was more obvious compared to group IV (1.5–2 log difference) (Figure 14). At the end of the experiment (day 14), the bacterial concentration in group I fish decreased by 2.7 L logs (9 × 10^3^ CFU/fish), while only 1.6 log decrease was observed in phage untreated fish group IV (Figure 14). The average daily mortality (13%) in group I was significantly (*p* < 0.05) lower compared to group IV (29%).

#### 2.4.3. Dynamic Changes in Abundance of Phages and Bacteria in Aquaria Water (Experimental Series 1)

**Figure 15:**
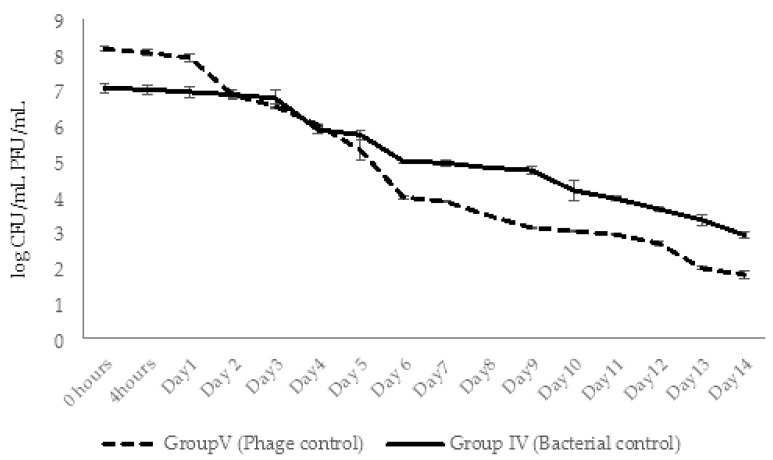
Dynamic changes in the concentration of AhMtk13a phage in aquaria water: Group V (phage control)—fish injected with saline and placed in the aquaria containing the phage only; Group IV (bacterial control)—fish injected with the *A. hydrophila* GW3-10 and kept in the aquaria containing the same bacterial pathogen. The results are the averages of three parallel experiments with geometric SD shown as the vertical lines.

After phage administration, the abundance of AhMtk13a phage in the aquaria of group V (phage control) remained stable only during 24 h. Gradual decline in the number of infective phage particles started at day 2 (decrease by 1.3 logs), continued until the end of the experiment (14 days), and finally resulted in a drop of phage particles by 6.3 logs (Figure 15). This significant (*p* < 0.05) decrease in viable phage counts seem to be related to the low stability of AhMtk13a phage in the tap water, which was shown in laboratory experiments (see Section 2.2.1).

The concentration of *A. hydrophila* GW3-10 in aquaria water (control group IV) maintained without significant changes during 3 days. Decline in bacterial counts started at day 4 and continued to decrease gradually during 14 days with the final 4 logs reduction (Figure 15).

**Figure 16:**
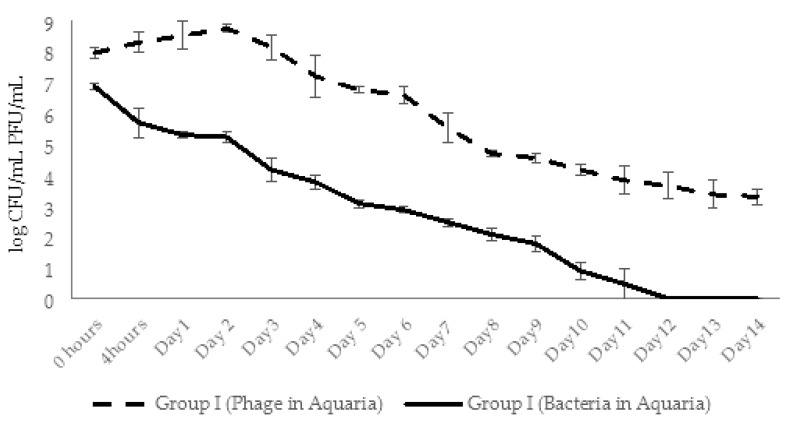
Dynamic changes in the concentration of AhMtk13a phage and *A. hydrophila* GW3-10 in aquaria water of experimental Group I—fish injected with *A. hydrophila* GW3-10, placed in the aquaria with the same bacteria added and immediately treated with the phage. The results are the averages of three parallel experiments with geometric SD shown as the vertical lines.

In experimental group I, for conditions of simultaneous administration of phage and bacteria at MOI 10:1, the phage numbers gradually increased by 1.1 log within 2 days and then started to decline gradually during 14 days. At the end of experiments the phage abundance in the aquaria water of group I decreased by 4.6 logs, but still maintained in slightly higher concentration (1.2 × 10^3^ CFU/mL) compared to control group V (6 × 10^2^ CFU/mL). In the same experimental group, the increase in phage numbers correlated with the decline in bacterial counts: after the 1 log decrease in bacterial counts in 4 h, the gradual decline continued until day 12 with the final 6.9 log reduction in bacterial concentration. By day 14 the bacterial counts were below detectable levels (Figure 16).

**Figure 17:**
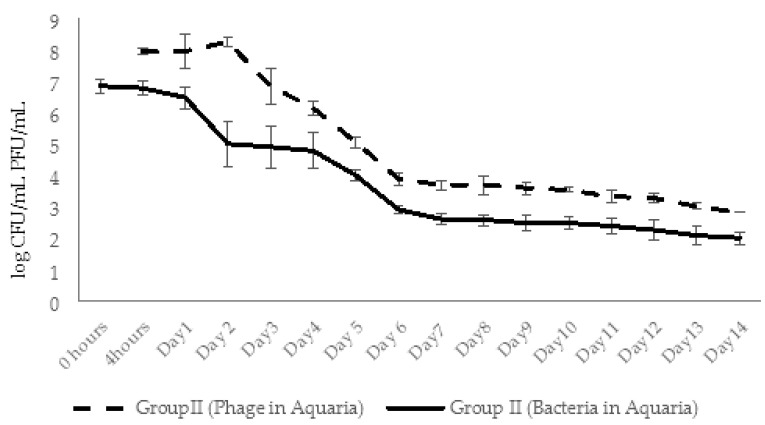
Dynamic changes in the concentration of AhMtk13a phage and *A. hydrophila* GW3-10 in aquaria water of experimental Group II—fish injected with *A. hydrophila* GW3-10, placed in the aquaria with the same bacteria added and treated with the phage in 4 h after infection. The results are the averages of three parallel experiments with geometric SD shown as the vertical lines.

In experimental group II, the zebrafish engrafted with x 10^7^ CFU/mL *A. hydrophila* GW3-10 were placed in the aquaria containing the same concentration of the pathogen. The phage AhMtk13a was added in 4 h to the water at the final concentration ×10^8^ CFU/mL (MOI 10:1). During the first 2 days, the phage abundance in aquaria water increased 4 times accompanied with 1.8 log decrease in bacterial counts (Figure 17). From day 3 the number of viable phage counts started to decline with parallel gradual decline in bacterial numbers. At day 6, concentration of phage and bacteria in aquaria water comprised ×10^3^ PFU/mL and ×10^2^ CFU/mL, correspondingly, followed by a plateau during next 7 days. Overall, during 14 days, the phage and bacterial abundance in group II aquaria decreased by 5.1 and 4.8 log, correspondingly, showing still with better survival rates compared to the control groups. 

**Figure 18:**
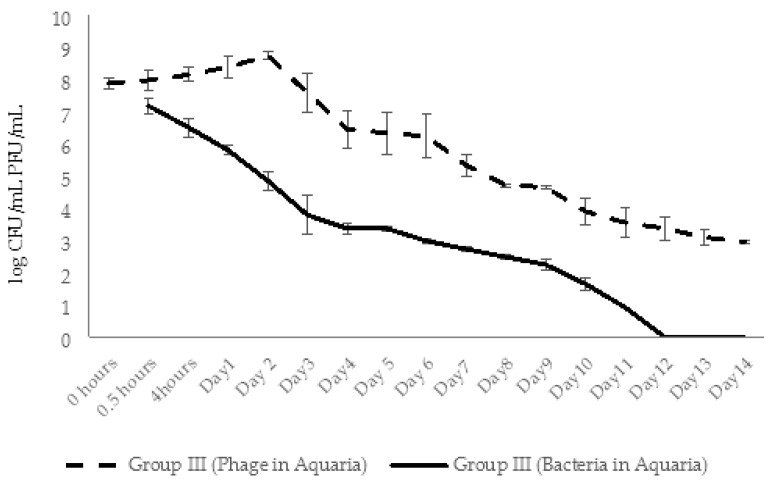
Dynamic changes in the concentration of AhMtk13a phage and *A. hydrophila* GW3-10 in aquaria water of experimental Group III—fish exposed to phage AhMtk13a containing aquaria 30 min before intraperitoneal injection with *A. hydrophila* GW3-10 and adding the same pathogen to the aquaria water. The results are the averages of three parallel experiments with geometric SD shown as the vertical lines.

In experimental group III, the phage AhMtk13a was added at the final concentration ×10^8^ PFU/mL to the aquaria containing intact zebrafish. After 30 min the fish was engrafted with the same pathogen (×10^7^ CFU/mL) and placed back in experimental aquaria to which *A. hydrophila* GW3-10 was added at the final concentration ×10^7^ CFU/mL. The two-fold increase in phage titer was already registered in 4 h (Figure 18). The number of infective phage particles continued to increase and reached maximal concentration (6 × 10^8^ PFU/mL) in 2 days after adding of the phage. This was in correlation with the decrease in bacterial counts, particularly: six-fold decrease in viable bacterial counts in 4 h, and 1.4 and 2.5 log decrease in 24 and 48 h, correspondingly. During days 3 to 8 the abundance of phages was gradually decreasing, but always exceeding the concentration of bacterial cells by ≥ 2 log in the same group. By day 10 the abundance of phages in group III aquaria reached the plateau and maintained in range of ×10^3^ PFU/mL, while the number of viable bacteria dropped below detectable levels. Finally, in 2 weeks the number of phages and bacteria in the water of group III aquaria was reduced by 5 and 7 logs, respectively (Figure 18).

### 2.5. Estimation of Antibacterial Efficacy of AhMtk13A Phage in the Experimental Zebrafish Model of A. hydrophila GW3-10 Infection: Experimental Series 2

In experimental series 2, conditions closer to naturally occurring processes were developed. Decreased concentration (×10^5^ CFU/mL) *A. hydrophila* GW3-10 was used in aquaria water and experimental fish were also engrafted with a reduced number of *A. hydrophila* GW3-10 (×10^5^ CFU/fish). The phage AhMtk13a was added to aquaria water at the final concentration ×10^7^ PFU/mL that resulted in increased phage: bacteria ratio 100:1. In addition, the phage was added to experimental aquaria three times: directly after bacterial challenge and on days 3 and 5 of the experiment. 

Two groups of zebrafish (each group consisted of 45 individual zebrafish equally divided into three parallels), group I (experimental) and group II (bacterial control), were engrafted with *A. hydrophila* GW3-10 and set to the aquaria containing the same pathogen. AhMtk13a phage was immediately added to group I aquaria. Group III (phage control)—fish engrafted with saline and exposed to the phage only, was monitored for assessment of phage maintenance in aquaria water and in fish, and to exclude the possible harmful effect of phages. Group IV fish were engrafted with saline (vehicle control).

#### 2.5.1. Protective Effect of AhMtk13a Phage Administration on Infected Zebrafish: Comparative Mortality Rates (Experimental Series 2)

**Figure 19:**
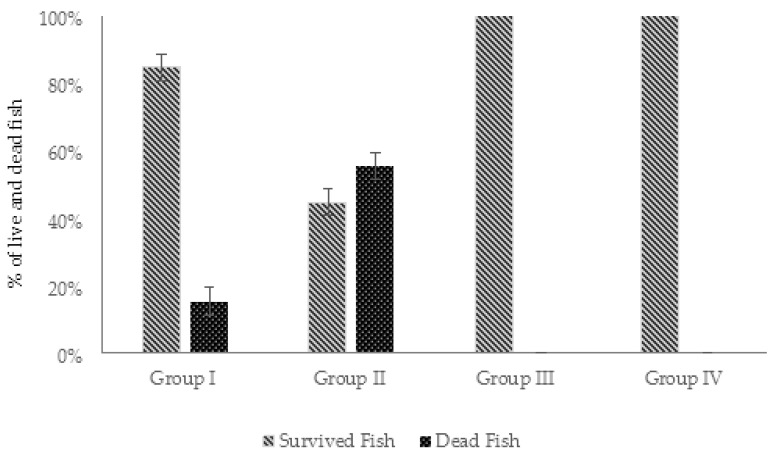
Protective effect of AhMtk13a phage on zebrafish infected with *A. hydrophila* 3-10. Survival and death rates (%) in different experimental groups: Group I—fish injected with *A. hydrophila* GW3-10, placed in the aquaria with the same bacteria added and immediately treated with the phage; Group II (bacterial control)—fish injected with the *A. hydrophila* GW3-10 and kept in the aquaria containing the same bacterial pathogen; Group III (phage control)—fish injected with saline and placed in the aquaria containing the AhMtk13a phage only; Group IV (vehicle control)—fish received saline only through intraperitoneal injection. Neither phage nor bacteria were added to these aquaria. The results are the averages of three parallel experiments with SD shown as the vertical lines.

The protective effect of AhMtk13a phage was monitored for 14 days after injection of the zebrafish with *A. hydrophila* GW3-10. The highest cumulative mortality (55%) in the infected zebrafish was registered in group II, which received no phage treatment (Figure 19). The zebrafish in this group started to die 4 h after bacterial challenge and reached the maximum daily mortality (26%) by end of the first day, while average daily mortality for 3 days comprised 19%. In group I, the fish mortality in all three parallels was registered on days 2 and 3 after bacterial challenge before adding the second phage dose to aquaria. The last single death in group I was observed on day 9. The average daily fish mortality in experimental group I comprised 5.5% and the average cumulative mortality, 15%, was significantly (*p* < 0.05) lower compared to group II. During the whole experiment, no mortalities were observed in experimental groups III (phage control) and IV (fish engrafted with saline) (Figure 19). 

The inoculated strain of *A. hydrophila* GW3-10 was isolated from three randomly selected individual fish from groups I and II. The average bacterial concentration in dead fish was ×10^7^ CFU/mL. All bacterial isolates expressed susceptibility to AhMtk13a phage.

#### 2.5.2. Dynamic Changes in Abundance of Phages and Bacteria in Zebrafish (Experimental Series 2)

The dynamic changes in the abundance of phage and host bacteria in zebrafish were studied in experimental group I and in control groups II and III. In total, 186 adult healthy zebrafish were used in challenge experiments according to the experimental protocols described in the Materials and Methods, Section 4.10 and Section 4.11.

**Figure 20:**
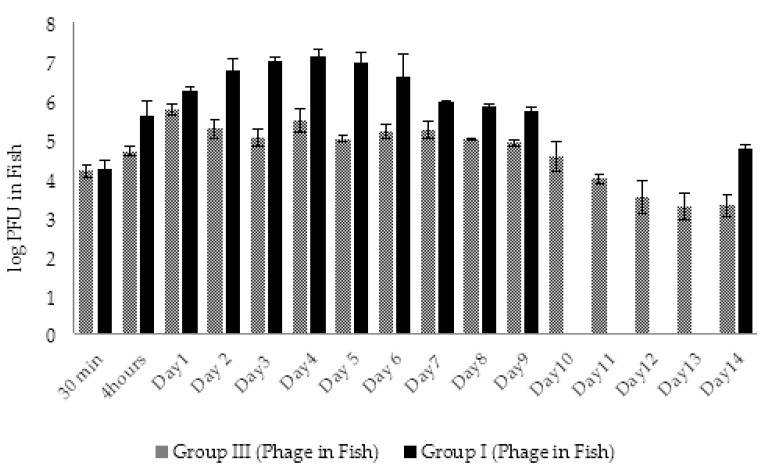
Dynamic changes in AhMtk13a phage concentration in phage-treated fish: Group I—fish injected with *A. hydrophila* GW3-10, placed in the aquaria with the same bacteria added and immediately treated with the phage; Group III (phage control)—fish injected with saline and placed in the aquaria containing the phage only. The results are the averages of three parallel experiments with geometric SD shown as the vertical lines.

In groups I and III, the phage AhMtk13a was already detected in fish 30 min after adding phage to the aquaria. The phage concentration in the examined fish from all parallels in both groups was in range of 1.5–1.8 × 10^4^ PFU/fish (Figure 20.). In the fish of group III, the number of bacteriophages increased by 1.6 logs in 24 h and after slight decrease in 48 h was maintained in the range of 1–3.5 × 10^5^ PFU/fish during next 7 days (Figure 20). A gradual decrease in phage concentration started from day 9 and at the end of the experiment the phage concentration in group III comprised 2.2 × 10^3^ PFU/fish, which is 2.5 log lower compared to the concentration at day 1 (Figure 20).

Unlike group III, the 1.4 log increase in AhMtk13a phage numbers was already observed in group I fish in the first 4 h (Figure 20). The highest phage concentration in fish was observed between days 3 and 5, comprising 1 × 10^7^ PFU/fish, which is 1.6–2 logs higher than in group III. The slow decline in phage numbers started from day 6 and by the end of the experiment the phage numbers were reduced to 4–7 × 10^4^ PFU/fish.

Thus, the AhMtk13a phage in fish of both groups was maintained throughout the experiment, with more obvious increase in phage titers in group I in the first 5 days. The concentration of AhMtk13a phage in the fish of group I always exceeded (by 5–100 times) the concentration of the same phage in group III. At the end of the experiment, the difference in the AhMtk13a phage counts between groups I and III comprised 1.4 log (Figure 20).

**Figure 21:**
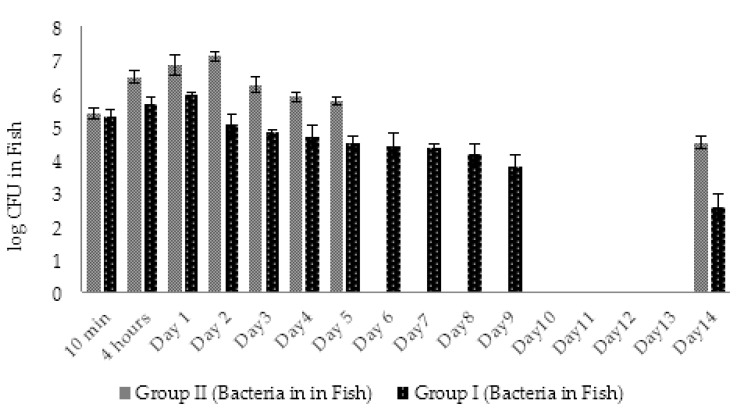
Dynamic changes in *A. hydrophila* GW3-10 concentration in fish: Group I—fish injected with *A. hydrophila* GW3-10, placed in the aquaria with the same bacteria added and immediately treated with the AhMtk13a phage; Group II (bacterial control)—fish injected with the *A. hydrophila* GW3-10 and kept in the aquaria containing the same bacterial pathogen. The results are the averages of three parallel experiments with geometric SD shown as the vertical lines.

The initial number of viable *A. hydrophila* GW3-10 cells in fish from groups I and II was in the range 2–2.4 × 10^5^ CFU/fish. Four hours after bacterial challenge, the concentration of bacteria in group II fish increased by 1 log and peaked on day 2 with the concentration 1.2 × 10^7^ CFU/fish (Figure 21). The gradual decrease in viable bacterial counts started at day 3 resulted in 1.8–4 × 10^4^ CFU/fish concentration at the end of the experiment. 

Different from control group II, the concentration of *A. hydrophila* GW3-10 in fish of group I (treated with phage) after the three times increase on day 1, started to decline continuously throughout the experiment, comprising 4.5 × 10^2^ CFU/fish on the last day of the experiment. On day 14, the number of bacteria in group I fish (experimental group) decreased by 2.7 logs, while only a 0.9 log decrease was registered in group II (bacterial control), where the highest fish mortality was recorded (Figure 19 and Figure 21). The decline in bacterial counts during days 2 to 5 correlated with the increase in phage numbers in fish of experimental group I (Figure 20 and Figure 21). 

#### 2.5.3. Dynamic Changes in Abundance of Phages and Bacteria in Aquaria Water (Experimental Series 2)

**Figure 22:**
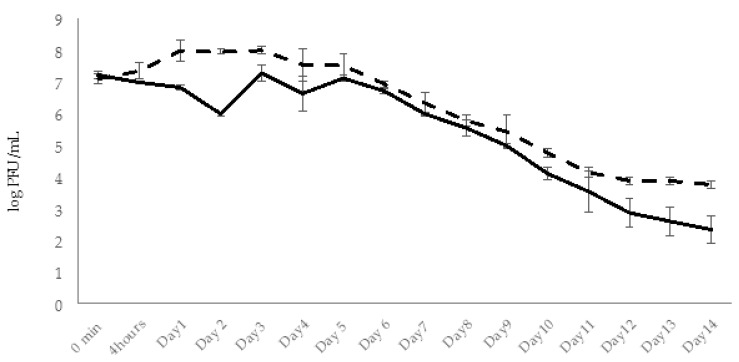
Dynamic changes in AhMtk13a phage survival in aquaria water: Group I—fish injected with *A. hydrophila* GW3-10, placed in the aquaria with the same bacteria added and immediately treated with the phage; Group III—fish injected with saline and placed in the aquaria containing the experimental phage only. The results are the averages of three parallel experiments with geometric SD shown as the vertical lines.

In group III (phage control) of experimental series 2, the AhMtk13a phage remained stable for 24 h after adding phage to aquaria (with final concentration ×10^7^ PFU/mL), followed by 1 log decrease in number of infective phage particles on day 2 (Figure 22). The increase in phage numbers observed on days 3 and 5 was due to adding repeated doses of the AhMtk13a phage to group III aquaria. A gradual decline in phage numbers in aquaria started from day 6, and continued until the end of the experiment (14 days). Finally, a 4.8 log drop in the number of infective phage particles was registered, comprising an average 292 PFU/mL in the aquaria water.

In group I aquaria, phage and bacteria were added simultaneously with the final concentrations 1.3 × 10^7^ PFU/mL and 4 × 10^5^ CFU/mL, correspondingly. The AhMtk13a phage numbers doubled in 4 h, increased 10 times in 24 h, and maintained practically in the same range (1.3 × 10^7^–1.1 × 10^8^ PFU/mL) during 5 days (Figure 22). The addition of the repeated phage doses on days 3 and 5 did not significantly change the final concentration of phage in aquaria water. The gradual decline in phage numbers started from day 6 and continued until day 12. For the last 3 days of the experiment, phage concentration reached a plateau and comprised ×10^3^ PFU/mL, being still 1.4 logs higher compared to group III (phage control).

**Figure 23:**
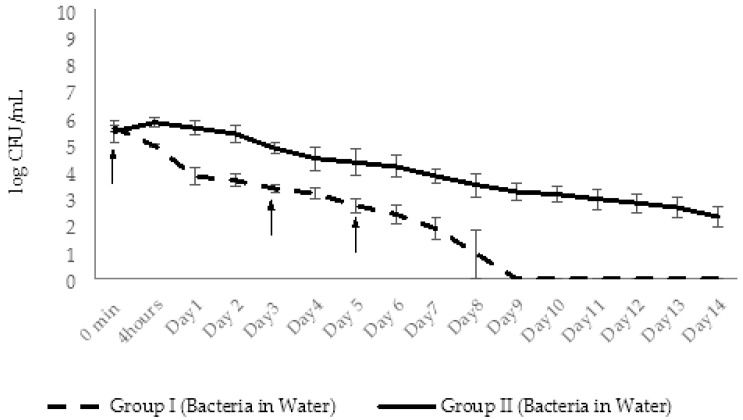
Dynamic changes in *A. hydrophila* GW3-10 counts in aquaria water: Group I—fish injected with *A. hydrophila* GW3-10, placed in the aquaria with the same bacteria added and immediately treated with the phage; Group II (bacterial control)—fish injected with the *A. hydrophila* GW3-10 and kept in the aquaria containing the same bacterial pathogen. The arrows indicate the application of AhMtk13a phage to group I aquaria. The results are the averages of three parallel experiments with geometric SD shown as the vertical lines.

The initial concentration of *A. hydrophila* GW3-10 (3.7 × 10^5^ CFU/mL), in group II aquaria (bacterial control) remained stable for 48 h, and then from day 3 started to gradually decrease until the end of the experiment (Figure 23.). On day 14, bacterial concentration comprised 2.5 × 10^2^ CFU/mL, which corresponds to 3.4 log reduction compared to the concentration at the starting point (Figure 23).

In contrast with group II, bacterial counts in aquaria water in group I decreased by 0.7 log in 4 h and by 2 logs in 48 h, comprising 4.8 × 10^3^ CFU/mL. After gradual decline during the next 6 days, the lowest countable average bacterial numbers (24 CFU/mL) were registered at day 8. From day 9, the number of viable bacteria in all parallels was below detectable levels (Figure 23).

## 3. Discussion

In the last decades, a rapid expansion in aquaculture production has been seen. It is expected that in the future farmed fish will become one of the most important affordable sources of protein foods worldwide [31]. Microbial diseases represent a large threat to aquaculture industries, frequently leading to heavy financial losses and affecting their growth and sustainability [32,33,34]. *Aeromonas hydrophila* as an aquatic bacterium often causes serious diseases in freshwater farmed fish. Control of this pathogen is complicated because of its natural multidrug resistance (MDR) [35,36,37]. Bacteriophages are considered a promising alternative means for control of fish microbial diseases. Phage therapy in medicine has a long successful history in Eastern Europe and FSU countries, including Georgia [38]. The results of the experimental studies with marine animal models demonstrated the efficacy of phage therapy against different infectious diseases [39,40,41]. The use of bacteriophage therapy for control of pathogenic *A. hydrophila* has also been reported [20,42].

In our study, we describe two new *Aeromonas*-specific bacteriophages—AhMtk13a and AhMtk13b—isolated from the Mtkvari River. The host bacterial strain *A. hydrophila* GW3-10 was isolated from diseased trout in one of the fish farms in Central Georgia. TEM examination of these two phage isolates showed that both belong to the order *Caudovirales*, with the phage AhMtk13a assigned to the family *Myoviridae* (with the elongated icosahedral head and contractile tail) and AhMtk13b assigned to family *Siphoviridae* (with the isometric icosahedral head and long noncontractile tail) [43]. The whole genome sequence analysis placed AhMtk13a within genera Tulanevirus, while phage AhMtk13b was identified as unclassified *Siphoviridae*. The AhMtk13a phage with genome size 163879 bp and CG content 41.21% showed high genome similarity (with average nucleotide identity (ANI) 95–97%) with six *A. hydrophila* phages (50AhdR13PP, Aes012, AS-gz, phiAS4, Aes508, 60AhydR15PP) and *Stenotrophomonas* phage IME13. The AhMtk13b phage with genome size of 61446 bp and CG content of 62.01% is homologous only with three *Aeromonas* phages available in the NCBI database (BUCT551, vB_AhyS-A18P4, and LAh_7) with high similarity only to *Aeromonas* phage LAh_7 (MK838113.1; ANI 95.94%). The AhMtk13a phage genome encodes 16 tRNAs, while no tRNA genes were found in the AhMtk13b phage genome. The number of tRNA genes in phage genomes may vary significantly. There are well-characterized phages without tRNA genes and phages with >40 tRNA genes [44]. The exact function of these genes is not completely clear. Supposedly, tRNA genes are involved in facilitating adaptation to new hosts, giving phages certain self-selected translational mechanisms, as deletion of tRNA has been found to reduce phage fitness [45]. 

Deep analysis of phage genome is important for selection of proper phages for therapeutic applications. No ORF encoding integrase was found in neither of the two new *A. hydrophila*-specific phage genomes, which is indicative of virulent nature of these bacteriophages [46,47]. It is commonly accepted by phage researchers and also strongly recommended by regulatory agencies for phage therapy purposes to use only virulent phages, as they do not risk entering a lysogenic cycle. The temperate phages should be avoided primarily because of possible development of lysogenic conversion that can lead to production of phage-encoded toxins and antibiotic resistance in bacteria [48,49].

One of the advantages of phage therapy versus antibiotics is its high specificity—the ability to destroy only target harmful bacteria without affecting the other beneficial bacteria in the body and in the environment [50]. The host range studies done for the newly isolated phages AkMtk13a and AhMtk13b have shown that they are species specific, lysing the strains of *A. hydrophila* only. The difference was revealed in lytic spectrum between these two phages: the broader host range (73% of tested strains) was shown for AhMtk13a phage that also covered the strains lysed by AhMtk13b phage. Based on the broad lytic spectrum of AhMtk13a phage, along with virion morphology (*Myoviridae* morphotype) and genomic characteristics (absence of integrase, presence of tRNAs), it was selected for further studies. 

Besides the host range for selection of phages for application in aquaculture, other characteristics should be considered, such as parameters of one cell growth cycle, survival in the environment, and antibacterial efficacy [51]. The Ahmtk13a phage demonstrated ability of fast adsorption (maximal adsorption in 10 min), short latent period (20 min), and the high burst size (160 phage particles per infected cell). These physiological parameters of AhMtk13a phage characterize it as a virulent phage with high activity toward the susceptible strain.

Estimation of phage lytic activity in liquid culture is important for selection of potential therapeutic phages in order to reveal the ability of a candidate phage to minimize the occurrence of phage-resistant bacterial mutants [52,53,54,55]. The phage AhMtk13a maintained lysis stability in liquid culture for 6 h at the lowest MOI (0.001), for 24 h at MOI 0.1, and for 48 h at MOI 1. This indicates the high potential of the phage AhMtk13a to be used as a therapeutic phage in liquid environment with low probability for development of phage resistance in fish pathogenic bacteria. 

The study of the phage sensitivity to external factors is considered especially important and useful for phage application in open environments such as agriculture, including aquaculture [56]. In our studies, AhMtk13a phage demonstrated high stability in PBS, saline, lake water, and in the fish farm water for 8 weeks. Less, but still sufficient stability of phage was registered in TSB and Black Sea water for the same time period. Our results differ from the data obtained by Kim et al. [57] on the stability of five Flavobacterium phages (PFpW-3, PFpC-Y, PFpW-6, PFpW-7, PFpW-8) isolated from Japanese ayu farm pond water. The phage survival (tested for 21 days at 18 °C) appeared to be much shorter with significant changes in the phage titer in pond water, decreasing below the detection limit after 10 days. In our study, AhMtk13a phage preserved the infectivity for a much longer time (≥8 weeks). It seems that the pattern of phage survival in the water environment may also vary based on the phage specificity. For example, in the study done by Carla Pereira et al. [58], *A. salominicida* phage AS-1 phage survived for a long period (91 days) in marine water, while the numbers of *V. parahaemolyticus* phage VP-1 decreased strongly during 16 days only. The AhMtk13a phage demonstrated less capability to survive in autoclaved tap water. The sharp decrease in phage infectivity started from the first week and by day 21 the number of viable phage particles was below the detection limit. A similar observation, described earlier by Jepson and March (2004) [59], was explained by presence of halogenating agents in tap water that may inactivate phages. The standard practice to use tap water for filling aquaria for zebrafish maintenance and reproduction can influence phage survival in aquaria water and, correspondingly, may reduce the positive outcome of phage treatment in our studies as well (see in Discussion below).

Temperature is a crucial factor for bacteriophage survivability and can also determine the occurrence and viability of bacteriophages, along with conditions for their storage [60]. Different from most bacteriophages with optimal temperatures in the range 15 to 40 °C, there are phages that may be resistant to low and high temperatures and settle in extreme environments [56], such as bacterial viruses isolated from hot springs in California. More than 75% of the phage particles remained intact even when incubated on ice (around 0 °C) and 18–30% of the phages remained intact at 105 °C [61]. The phages specific to aquatic bacteria respond differently to changes in surrounding environment, including temperature increase or decrease. In the experimental studies done by Muhamad Akmal et al. [18], survival of *A. hydrophila* phage Akh-2 was shown to be 100% between −80 and 37 °C, but no active phage was observed after 3 days incubation at 55 or 60 °C. Our investigations did not show any reduction in phage titer after storage at the ambient temperature, at 30 °C, and at 37 °C during one week. There was no reduction in phage titer after incubation of AhMtk13a phage at 40 and 50 °C for 1 h. However, elevated temperatures (60 and 70 °C) seriously influenced AhMtk13a phage infectivity, decreasing the number of intact phage particles in 1 h by 4.4 and 6.6 logs, correspondingly.

Another important factor influencing phage stability is the acidity of the environment. The studies done on stability of phages in acidic and alkaline environment during different time periods (from 1 h to 3 weeks) showed that the physical stability of phage was mainly in range of pH 5 to 9, almost completely losing the infectivity at the pH ≤ 3 and ≥ 11 [59,62,63]. Our own studies on the stability of AhMtk13a bacteriophage at different pH (from 2 to 12) during 1 h showed that phage preserves high survival rates in range of pH 4–10, while at pH 2 and 12, a decrease in the viable counts by 3 and 6 logs, correspondingly, was observed. Since in most Georgian fish farms the water pH is in the range 6–8, the activity of AhMtk13a phage is not expected to be reduced. 

After detailed phenotypic and genetic characterization of phage AhMtk13a, we performed a series of experiments for assessing its therapeutic potential to control *A. hydrophila* infection in zebrafish. The continuous infection in fish in our studies was achieved by a combination of two routes: intraperitoneal injection of fish pathogenic strain *A. hydrophila* GW3-10 and by applying the pathogen directly to experimental aquaria. In some of previous studies phages were administered via fish feed and injection [64,65,66,67], which was considered not very appropriate by other scientists who preferred the methods of phage administration directly to water [68,69]. Using this approach, Laanto et al. observed increase in zebrafish survival that was most likely due to a decrease in bacterial infection dose when the phage was added into aquaria directly after bacteria [68].

In our study we selected the method of direct application of experimental phage to aquaria in order to reduce the pathogen insemination in water and the possibility of the pathogen transmission through the water. Mini environment of fish aquaria also provided proper conditions for studying phage–bacteria interaction by using different regimens of phage administration. In particular, we evaluated the effect of administering three phage doses on the survival of infected zebrafish and to compare to the outcome of administering a single dose. In experimental series 1, a significant (*p* < 0.05) increase in the survival of zebrafish was observed in the phage-treated groups I and III (56% and 53%, correspondingly) compared to control group IV (22%). These results were obtained after the single dose phage application at MOI 10:1 simultaneously with bacterial challenge (group I), or 30 min before bacterial challenge (group III). The results of this part of experimental series 1 are in certain agreement with the fish survival data obtained by Akmal et al. [18] in studies conducted on *A. hydrophila* phage Akh-2. In our studies involving AhMtk13a phage, considerably lower survival rates (38%) but still better compared to control group were registered in the fish treated with phage at 4 h post infection (group II). The fish in this group, as in the control group, began to die already in 4 h after the bacterial challenge—much earlier than in groups I and III (24 h). Based on the results obtained, we can assume that phage administration order and time seem to play an important role in the outcome of phage treatment in fish. Delayed application of phage (that may reflect the real situation in fish farms) still can provide protection and reduce the mortality in infected individuals. The application of several doses of AhMtk13a phage at MOI 100:1 (in experimental series 2) resulted in better survival rates (85%) in infected fish, compared to the bacterial control group (45%). The additional factor—higher phage–bacteria ratio, 100:1—should be taken into consideration as well. We cannot provide here the analysis of other studies and compare with the results of our experiments on efficacy of multiple administrations of therapeutic phages in model fish simply because such regimen of phage treatment has not been used by other researchers (at least not found in literature sources available to us). It should be also mentioned that other details of experimental design (e.g., concentration of bacteria and phages, MOI, route of infection, etc.) may differ significantly between the particular studies targeting phage-based control of fish bacterial diseases. 

Another outcome of our experiments was the knowledge gained concerning the dynamic interaction of phages and bacteria in the process of phage-based treatment of fish bacteriosis in zebrafish model experimental conditions of fish aquaria. The experiments on phage–bacterial interaction in aquaria water showed that the addition of the AhMtk13a phage to water before and simultaneously with bacteria effectively eliminated the pathogen from the water environment. We were interested in quantitative distribution of AhMtk13a phage particles in zebrafish during the passive immersion in aquaria water containing phage and its host bacteria, i.e., in the conditions that may mimic the natural situation in fish ponds. It appeared that quite high concentration (×10^4^–×10^5^ PFU/fish) of phage can be observed in fish shortly (in 30 min) after the phage administration, reaching its maximum in 48 h and maintained throughout the experiment. The more obvious increase in phage titers was registered for the infected fish. It should be mentioned that the AhMtk13a phage concentration in the infected fish always exceeded the concentration in control groups (fish exposed to phage only) in both series of experiments. Our results indicate the fast and efficient penetration of phages into fish body from the water environment that guarantees a sufficient phage concentration to resist the infection by bacterial pathogen. Thus, there is no need for injections, especially when handling large numbers of fish. The active propagation of the AhMtk13a phage on pathogen cells within fish body is evidenced by the significantly (*p* < 0.05) higher (2 log) numbers of phage in fish of the experimental groups compared to the phage control groups. Interestingly, AhMtk13a phage was better preserved in fish than in water: the titer of phage in fish 10 times exceeded the titer of phage in water at the end of the experiments. This fact together with the high probability of phage persistence in aquaria water and fish body in sufficiently high concentrations during at least 10 days can provide good perspective for prophylactic use of phages in fish farms, especially during expected seasonal outbreaks of fish diseases caused by *A. hydrophila*. In the next step of our studies—series 3 of model experiments—we plan to use the mixture of AhMtk13a phage with two or more other selected phages with broad overlapping lytic spectrum. The use of combined phage preparation in conjunction with application of multiple phage doses can significantly increase the efficiency of phage treatment for control of *A. hydrophila* infections in farmed fish.

## 4. Materials and Methods

### 4.1. Bacterial Strains

Forty-nine *Aeromonas* strains were used in this study: 46 Georgian strains, majority collected from sick fish and water in 5 fish farms in Central Georgia, including 39 strains of *A. hydrophila*, 5 strains of *A. caviae*, and 2 strains *A. sobria* (collection of the Eliava Institute. 3 Gotua street, 0160 Tbilisi, Georgia); 3 reference strains: *A. hydrophila* CIP103770, *A. salmonicida achromogenes* CIP 104001T, and *A. salmonicida salmonicida* CIP 103209T, obtained from Collection of Institute Pasteur (Collection De L’Institut Pasteur (CIP), 28 rue du Docteur Roux 75724 Paris CEDEX 15). For the detailed list of strains, see Tables S1 and S2.

For propagation of bacterial strains and for testing of phage activity, Tryptic Soy Broth (TSB) and Tryptic Soy Agar (TSA) (Liofilchem, Italy) were used. The characteristic cultural properties of *Aeromonas* strains were checked on *Aeromonas* Selective Agar (ASA) (Liofilchem, Italy). 

### 4.2. Model Organisms

Zebrafish (*Danio rerio*) was selected as a model organism for the studies involving fish pathogenic bacteria and specific phages. The breeding of zebrafish populations for use in experiments was conducted in standard conditions in aquaria facilities of the Black Sea Flora and Fauna Research Center (BSFFRS) in Batumi, Georgia. Zebrafish were fed with *Artemia* sp., produced in-house at the BSFFRS. In total, 1162 zebrafish individuals twelve weeks of age were used in the studies. Particularly, 300 zebrafish were used for the estimation of fish mortality dependence on pathogens and 40 used for estimation of possible harmful effect of phage on zebrafish. In the experimental studies on antibacterial efficacy of phages (series 1 and 2), 822 zebrafish individuals were used. 

### 4.3. Isolation of A. hydrophila—Specific Bacteriophages

The water from the Mtkvari River was enriched with the *A. hydrophila* GW3-10 using the following mixture: 10 mL of 10× TSB, 1 mL of bacterial culture (×10^8^ CFU/mL in TSB), and 100 mL river water prefiltered with 0.45 µm cellulose acetate membrane filters (VWR). The enriched sample was incubated at 30 °C for 18 h, 800 µL of chloroform (Sigma-Aldrich) was added to the sample, mixed, and stored in a refrigerator for 1 h. The sample was centrifuged at 4000 rpm for 20 min and filtered through a 0.45 µm membrane filter. 

The enriched water samples were checked for the presence of phages by phage spot test using the parallel streaks method [70]. Briefly, a sterile cotton swab was dipped into bacterial suspension (×10^8^ CFU/mL in TSB) and applied in horizontal strips to a Petri dish with 1.6% (*w*/*v*) TSA. The strip was air-dried and spotted with 10 µL of test sample. After incubation for 18 h at 30 °C, the plates were observed for the presence of clear zones or plaques at the spotted points. 

For the observation of phage negative colonies, the soft agar overlay method was used [23] by mixing of 100 μL of exponential-phase bacterial culture with 1 mL of enriched water sample and 3 mL of soft agar (0.6% *w*/*v* agar) and poured onto TSA plates. After incubation at 30 °C for 18–24 h, the plates were examined for the presence of phage plaques.

### 4.4. Purification and Propagation of Phages

To obtain pure phage lines, the phage plaques with different morphology were picked up with a sterile pipette tip, inoculated into 1 mL of sterile TSB, and incubated at 30 °C for 2 h [23]. Ten-fold dilutions (from 10^−1^ to 10^−4^) were prepared in the tubes with TSB and used for obtaining plaques by the soft agar overlay method [23]. Plates containing 10–25 plaques were used for the next purification cycles (≥5 in total) until homogeneous plaques were obtained for each phage isolate. High titer bacteriophage stocks for each pure phage line were prepared by the soft agar overlay method [23]. The upper layer (0.6% *w*/*v* TSA) from the plates with semiconfluent lysis was collected using sterile spreaders after adding 2 mL TSB to each plate and centrifuged at 6000 rpm for 30 min. The supernatant was filtered through 0.45 μm membrane filters and phage titer was determined by soft agar overlay method. 

The concentrated phage suspensions (average titer 5 × 10^10^ PFU/mL) were further purified by following procedure: ≥20 mL of phage suspension in TSA was sedimented by centrifugation at 37,000× *g* for 1.5 h at 4 °C (Allegra64R, Beckman Coulter, IN, USA). The supernatant was discarded and 1 mL phosphate buffer saline (PBS) was added to the pellet. The centrifuge tubes were stored at 4 °C overnight and then centrifuged at 6000 rpm for 10 min. The average titer of obtained phage preparation comprised 1–5 × 10^11^ PFU/mL. 

### 4.5. Characterization of Bacteriophages

#### 4.5.1. Phage Virion Morphology

Transmission electron microscopy (TEM) was used to study the nucleocapsid morphology of the bacteriophages as described in [71] with some modifications. Three hundred-mesh Cooper grids with formvar/carbon films (FCF300-CU, Electron Microscopy Sciences, Fort Washington, PA, USA) were floated face-down on a 10 µL droplet of a purified phage sample (5 × 10^10^ PFU/mL in PBS) for 10 min at room temperature, washed with deionized water, treated with 2% uranyl acetate for 2 min, and examined in an electron microscope 100SX (Jeol, Akishima, Japan) at 80 KV and the instrumental magnification of 50,000. Ten phage particles for each phage were measured and the average size of head and tail was calculated.

#### 4.5.2. Phage DNA Extraction

For isolation of DNA from bacteriophages, the concentrated phage suspensions with the titer 1 × 10^11^ PFU/mL were used. DNA was extracted using QIAamp DNA Mini and Blood Mini kit (QIAGEN, Hilden, Germany) according to the manufacturer’s instructions. The DNA concentration was measured using microvolume spectrophotometer Nano Drop One (Thermo Scientific, Waltham, MA, USA).

#### 4.5.3. Bacteriophage Whole Genome Sequencing and In Silico Analysis

DNA sequencing libraries were prepared using the NEBNext Ultra DNA library prep kit. Sequencing was performed on the Illumina MiSeq Dx instrument using RUO (research use only) mode, using the v2 500-cycle sequencing chemistry. Illumina read quality was assessed using FastQC v0.11.7; de novo assembly was performed using CLC Genomics Workbench v12.0.3, with resulting average depth of read coverage 925x for AhMtk13a and 518x for AhMtk13b. Assembled phage genome was annotated manually using the Artemis annotation tool [72]. The putative open reading frames (ORFs) were predicted by using GenemarkS [73]. Putative functions of the ORFs were analyzed by BLASTP search and Pfam [74], and HMMER online software [75]. Prediction of tRNAs was done by using tRNAscan-SE 1.3.1 software [76]. Geneious software was used for multiple alignments and mapping [77]. Phage taxonomy was determined by using BLASTn results and was visualized by VIPtree [24,78]. Genome sequence identity was calculated by ANI Calculator based on the OrthoANIu algorithm [79]. Phylogenetic tree was constructed by MegaX with 500 bootstrap (neighbor-joining tree) [80]. GenBank accession number for our nucleotide sequence: BankIt2526887 *Aeromonas* OL840900 for Phage AhMtk13a; BankIt2529090 *Aeromonas* OL840901 for Phage AhMtk13b.

#### 4.5.4. Phage Host Range 

The phage suspensions (×10^8^ PFU/mL) were spotted on parallel streaks of bacterial culture on TSA plates and incubated for 18–24 h at 30 °C. The lysis intensity was registered as: CL, confluent lysis; SCL, semiconfluent lysis; OL, overgrown lysis; IP, individual plaques; and R, resistan [70]. 

#### 4.5.5. Phage Adsorption and One-Step Growth Cycle

Nine hundred μL of exponentially grown culture of *A. hydrophila* GW3-10 (1 × 10^8^ CFU/mL in TSB) was mixed with 100 μL of phage (1 × 10^8^ PFU/mL in TSB) and incubated at 30 °C. Samples (100 μL) were taken from the adsorption tube every 2 min (during 30 min), diluted 100 times in TSB + 0.4 mL chloroform, kept on ice for 10 min, and the tube contents titrated by the double layer method to determine the number of infective phage particles. The percent and time of maximal adsorption were determined according to the method described by Adams [23].

In the one-step growth experiment, bacterial suspension (×10^8^ CFU/mL) was mixed with the phage at the MOI of 0.1. The adsorption process was allowed to occur during 10 min at 30 °C in water bath. The mixture was centrifuged at 13,000× *g* for 1 min, the pellet (phage-infected cells) was resuspended in 10 mL of TSB and incubated at 30 °C for 80 min. The samples were taken at 5 min intervals and the number of infected phage particles determined by the soft agar overlay method [23]. The latent period was defined as the interval between maximal adsorption of the phages to the bacterial cells and the beginning of the release of phage progeny [81]. The burst size of the phage was calculated according to [82].

#### 4.5.6. Lysis Stability of AhMtk13a in Liquid Culture

The lysis stability of AhMtk13a phage was studied in TSB on the host strain *A. hydrophila* GW3-10 according to the method of Appelmans [26]. Briefly, serial 10-fold dilutions (from 10^−1^ to 10^−7^) of phage with initial concentration of 10^9^ PFU/mL were prepared by transferring 0.5 mL of phage suspension to the tubes with 4.5 mL TSB. *Aeromonas hydrophila* GW3-10 was grown in TSB to the exponential phase, diluted 10 times, and 50 μL was added to all tubes with phage dilutions with a final concentration of 10^5^ CFU/mL, resulting in the multiplicity of infection (MOI) ranging from 1000 to 0.001. Phage lytic activity was evaluated visually by examining bacterial growth (turbidity) at 6, 24, and 48 h, comparing with McFarland turbidity standard (MFTS) tubes (McFarland Standards Kit, bioMerieux).

### 4.6. Stability and Infectivity of Bacteriophage AhMTK13a in Different Environmental Conditions

#### 4.6.1. Survival in Different Liquid Environment

The stability of AhMtk13a phage to various solutions and natural waters (saline, PBS, TSB, filter sterilized and autoclaved lake water, Black Sea water, fish farm water, and tap water) was monitored on a weekly basis for 8 weeks. AhMtk13a phage (2 mL) was added to 50 mL media bottles containing 18 mL of the different liquids so that the final concentration of phage comprised ×10^8^ PFU/mL. The bottles were kept at room temperature. One milliliter sample from each bottle was taken every week and the number of active phage particles determined by the soft agar overlay method [23].

#### 4.6.2. Influence of Temperature and Different pH on Survival AhMtk13a Phage

Reaction tubes with phage (×10^8^ PFU/mL) were incubated at various temperatures (40, 50, 60, and 70 °C). Samples were collected at 15, 30, and 60 min to determine the number of infective phage particles by the soft agar overlay method [23].

To test the influence of pH, SM buffer was adjusted to different pH (2, 4, 6, 8, 10, and 12). The phage was inoculated to a final concentration of ×10^8^ PFU/mL and incubated at 30 °C. Samples were taken at 30 and 60 min to determine the number of infective phage particles by the soft agar overlay method [23].

### 4.7. Assessment of Bacterial Pathogenicity In Vivo: Development of Infection Model for A. hydrophila GW3-10

#### 4.7.1. Estimation of Zebrafish Mortality Depending on the Concentration of *A. hydrophila* GW3-10

All experiments were conducted in 5 L aquaria equipped with oxygen supplier and filled with 2.5 L of dechlorinated tap water. In order to make them more susceptible to the infection, the food supply was cut 1 day prior to the start of the experiments [28]. 

Exposure by immersion: The experiments were designed as described in Jun Xie et al. [28] with some modifications. Briefly, for each bacterial dose, with final concentration ×10^5^ and ×10^7^ CFU/mL, 4 aquaria were used (3 experimental parallels and 1 control). The biomass *A. hydrophila* GW3-10 for inoculation was obtained from the overnight culture on TSA agar slants washed with sterile saline 3 times. The suspension obtained was added to 3 experimental aquaria. Thirty zebrafish individuals, divided into 3 groups (10 per aquaria) were immersed in the aquaria containing *A. hydrophila* GW3-10. Group 4 with 10 zebrafish was kept in a separate aquarium (with no bacteria added). Zebrafish were observed for 14 days.

Exposure by immersion following dermal abrasion: Forty zebrafish were anesthetized using 160 mg/mL tricaine, MS-222. Each anesthetized zebrafish was scraped along the lateral surface behind the pectoral fins with a sterile scalpel to remove several scales and scratch the underlying dermis with a technique described by Neely et al. [83,84]. Three groups of zebrafish (10 per aquaria) were immersed in aquaria with *A. hydrophila* GW3-10 at final concentration ×10^7^ CFU/mL. The fish of group 4 (10 individuals) were then kept in separate aquaria (control group). Zebrafish were observed for 14 days.

Exposure by intraperitoneal injection: The experiments were performed as described by Phelan et al. [85]. Briefly, 180 zebrafish individuals anesthetized with 160 mg/mL tricaine (MS-222) were divided into 6 groups (in 3 parallel aquaria in each group, containing 10 individuals per aquaria). The zebrafish of the 5 groups were injected intraperitoneally with *A. hydrophila* GW3-10 at different increasing concentrations (×10^3^, ×10^5^, ×10^6^, ×10^7^, and ×10^8^ CFU/mL) and placed in the aquaria containing ×10^7^ CFU/mL *A. hydrophila* GW3-10. Group 6, engrafted with sterile saline, were placed in 3 separate aquaria with no added bacteria (control group). Zebrafish were observed for 14 days.

#### 4.7.2. Estimation of Safety of Treatment with Phage AhMtk13a on Zebrafish

In order to exclude the possible harmful effects of bacteriophage AhMtk13a on zebrafish, in situ phage assessment was performed. This included the application of AhMtk13a phage to aquaria with zebrafish.

To exclude the presence of viable bacterial cells in AhMtk13a phage preparation, before the addition of phage to the aquaria, the phage preparation was filter-purified using 0.22 µm sterile syringe filters, spread on ASA plates, and incubated overnight at 30 °C. The absence of bacterial growth was considered as indicator of sterility of phage preparation. 

Forty zebrafish individuals were equally divided into four aquaria, each containing 10 fish individuals. The phage, with final concentration ×10^8^ PFU/mL, was administered to three aquaria. One aquarium served as a control and received 2 mL of sterile saline. Zebrafish in aquaria were monitored during 14 days for visible physical and behavioral parameters (overall body condition, swimming behavior) and also for signs of possible sickness. 

### 4.8. Estimation of Antibacterial Efficacy of AhMtk13A Phage in the Laboratory Conditions in Aquaria Water and in the Zebrafish (Experimental Series 1)

#### 4.8.1. Protective Effect of AhMtk13a Phage Administration on Infected Zebrafish

All experiments were conducted in triplicates. Six experimental groups were created:

1. Group I—fish injected with *A. hydrophila* GW3-10, placed in the aquaria with the same bacteria added, and immediately treated with the phage;
2. Group II—fish injected with *A. hydrophila* GW3-10, placed in the aquaria with the same bacteria added, and treated with the phage in 4 h after infection;
3. Group III—fish exposed to phage AhMtk13a containing aquaria 30 min before intraperitoneal injection with *A. hydrophila* GW3-10 and adding the same pathogen to the aquaria water;
4. Group IV (bacterial control)—fish injected with the *A. hydrophila* GW3-10 and kept in the aquaria containing the same bacterial pathogen;
5. Group V (phage control)—fish injected with saline and placed in the aquaria containing the experimental phage only;
6. Group VI (vehicle control)—fish received saline only through intraperitoneal injection. Neither phage nor bacteria were added to these aquaria;

Each group consisted of 45 fish individuals equally distributed in the 3 aquaria (15 individuals per aquarium). 

Experimental fish in groups I, II, III, and IV were engrafted with *A. hydrophila* GW3-10 at the concentration ×10^7^ CFU/mL using the method described above (see in Methods, Section 4.7) and set to the experimental aquaria containing the same bacterial pathogen at the final concentration, ×10^7^ CFU/mL. 

The experimental phage AhMtk13a phage was added to the aquaria of groups I, II, III, and V at the final concentration, ×10^8^ PFU/mL.

Zebrafish health status together with phage and bacterial concentration in water were monitored daily during 14 days.

#### 4.8.2. Enumeration of AhMtk13a Phage and *A. hydrophila* GW3-10 in Aquaria Water 

The 1 mL water samples were collected from all aquaria of selected groups. For enumeration of bacteria in the aquaria water, serial (ten-fold) dilutions were prepared and spread plate technique was performed on the plates with ASA. For enumeration of phages in water samples from aquaria of the selected groups, the soft agar overlay method was used [23].

### 4.9. Enumeration of AhMtk13a Phage and A. hydrophila GW3-10 in Zebrafish

Dynamic changes in quantity of bacteria and phage in zebrafish was studied in group I and in control groups IV and V. To each parallel aquarium in groups I and IV (see Methods, Section 4.8.1) with 15 fish, 1 fish was added to be examined in 10 min after bacterial challenge for estimation of bacterial concentration in fish body. 

Three fish individuals (1 from each parallel) of group V were examined daily on presence of AhMtk13a phage during all 14 days. The fish in groups I and IV were monitored daily on presence of both phage and bacteria in all parallels during the first 5 days and on the last day, the 14th, of the experiment. Examination of fish on selected days was done because of gradually decreasing fish numbers in aquaria due to the fish mortality. 

For sacrifice the animals/fish were over-anaesthetized with tricaine methanesulfonate (MS-222). The fish were washed with sterile distilled water to remove the phages and bacteria from the fish surface. Whole fish were homogenized (each individual separately) using a 50 mL disposable tissue grinder (Fisherbrand™, Hampton, NH, USA) [86]. Serial ten-fold dilutions of each fish homogenate were prepared in sterile saline. Spread plate technique was performed on ASA plates for enumeration of *A. hydrophila* GW3-10. The soft agar overlay method was used for enumeration of AhMtk13a phage [23].

### 4.10. Estimation of Antibacterial Efficacy of AhMtk13A Phage in the Laboratory Conditions in Aquaria Water and in the Zebrafish (Experimental Series 2)

Similar to the description above (see Methods, Section 4.8), all experiments were conducted in triplicates, with some modifications. Briefly, 4 experimental groups of 45 fish individually set in 3 parallel aquaria were created.

1. Group I—fish injected with *A. hydrophila* GW3-10, placed in the aquaria with the added same bacteria, and immediately treated with the phage;
2. Group II (bacterial control)—fish injected with the *A. hydrophila* GW3-10 and kept in the aquaria containing the same bacterial pathogen;
3. Group III (phage control)—fish injected with saline and placed in the aquaria containing the experimental phage only;
4. Group IV (vehicle control)—fish received saline only through intraperitoneal injection. Neither phage nor bacteria were added to these aquaria.

A decreased concentration of *A. hydrophila* GW3-10 in aquaria water (×10^5^ CFU/mL) was used. AhMtk13a phage was added to the aquaria of group I and III at the final concentration ×10^8^ PFU/mL, which made up the ratio of phage–bacteria in aquaria 100:1. The phage was added to group I and III aquaria directly after bacterial challenge and on days 3 and 5 after starting the experiment. The experimental fish was engrafted with ×10^6^ CFU/mL *A. hydrophila* GW3-10. Zebrafish, along with phage and bacterial concentration in water, were monitored daily during 14 days. For enumeration of viable bacteria and infective phage particles in aquaria water, the methods described above (see Methods, Section 4.8.2) were used.

### 4.11. Enumeration of AhMtk13a Phage and A. hydrophila GW3-10 in Zebrafish (Experimental Series 2)

Dynamic changes in numbers of bacteria and phage in zebrafish were studied in experimental group I and in control groups II and III (see Methods, Section 4.10). One additional fish from each parallel aquaria of groups I and II was examined in 10 min after bacterial challenge to estimate the bacterial concentration in fish body after injection of *A. hydrophila* GW3-10. 

One fish from each parallel of group III (3 fish per group) was monitored daily on presence of AhMtk13a phage during all 14 days. The fish in groups I and II were examined daily to determine phage and bacteria counts during days 1–9 and days 1–5, correspondingly, and then on day 14. For enumeration of infective phage particles and viable bacterial cells in fish, the methods described in Section 4.9 were used. 

### 4.12. Statistical Analysis

All tests were carried out in triplicate for each sample and the numbers were averaged to create a single value for each variable. Mean value and standard deviations were calculated using Microsoft Excel 2017. Single factor analysis of variance (ANOVA) was conducted using the Statistical Tool Pak for Microsoft Excel 2017. A *p*-value < 0.05 was considered as statistically significant.

## 5. Conclusions

The new phages AhMtk13a and AhMtk13b, isolated on the fish pathogenic *A. hydrophila* GW3-10, are lytic bacteriophages based on genome characteristics. AhMtk13a belongs to the *Myoviridae* morphotype and AhMtk13b to *Siphoviridae* morphotype. AhMtk13a phage has broad host range, high lytic activity in liquid culture, and maintains the viability in different environmental conditions, including those of warm- and cold-water fish farms. Experimental studies on model zebrafish showed that AhMtk13 phage is safe for fish, does not cause fish mortality, and effectively penetrates the fish body from the water environment rapidly, reaching and maintaining the sufficient concentrations to resist the bacterial pathogen. AhMtk13a can significantly decrease the numbers of *A. hydrophila* in infected fish and aquaria water and therefore has potential to be used as a therapeutic and preventive tool in aquaculture. 

## Figures and Tables

**Table 1:** Lytic activity of bacteriophages AhMtk13a and AhMtk13b.

Bacterial Strains	Total Number of Isolates	Susceptible to Phage AhMtk13a	Susceptible to Phage AhMtk13b
*A. hydrophila*	39	28/39	13/39
*A. hydrophila* CIP103770	1	1/1	0/1
*A. caviae*	5	0/5	0/5
*A. sobria*	2	0/2	0/2
*A. salmonicida* CIP 104001T; CIP 103209T	2	0/2	0/2

**Table 2:** Lysis stability of AhMtk13a in liquid culture (according to the method of Appelmans).

Control *A. hydrophila* GW3-10 in TSB	MOI of AhMtk13a and *A. hydrophila* GW3-10 in TSB							
	0.001	0.01	0.1	1	10	100	1000	
0.5 MFTS *	^_^	^_^	^_^	^_^	^_^	^_^	^_^	6 h
2 MFTS *	1 MFTS *	0.5 MFTS *	^_^	^_^	^_^	^_^	^_^	24 h
3 MFTS *	2 MFTS *	1 MFTS *	0.5 MFTS *	^_^	^_^	^_^	^_^	48 h

* McFarland turbidity standard (MFTS); 0.5 MFTS corresponds to 1.5 × 10^8^ CFU/mL, 1 MFTS corresponds to 3 × 10^8^ CFU/mL, 2 MFTS corresponds to 6 × 10^8^ CFU/mL, 3 MFTS corresponds to 9 × 10^8^ CFU/mL, Average results of three parallel experiments.

## Data Availability

The data presented in this study are available in the article and in Supplementary Materials.

## Supplementary Materials

The following supporting information can be downloaded at: https://www.mdpi.com/article/10.3390/v14020412/s1, Table S1: *Aeromonas* Strains from Collection of G. Eliava Institute of Bacteriophages, Microbiology and Virology, Table S2: Lytic activity of Bacteriophage AhMtk13a and AhMtk13b.
