# Supplementary material for: Phenotypic and Genetic Characterization of Aeromonas hydrophila Phage AhMtk13a and Evaluation of Its Therapeutic Potential on Simulated Aeromonas Infection in Danio rerio
Source: Viruses. 2022 Feb 17;14(2):412. doi: 10.3390/v14020412 (PMC8876716; doi:10.3390/v14020412)
Supplement: Supplementary file 1 [file viruses-14-00412-s001.zip › viruses-1555434-supplementary.pdf]

Supplementary Materials:

GenBank accession number for our nucleotide sequence:

BankIt2526887 Aeromonas OL840900 for Phage AhMtk13a

BankIt2529090 Aeromonas OL840901 for Phage AhMtk13b

Table S1: *Aeromonas* Strains from Collection of George Eliava Institute of Bacteriophages, Microbiology and Virology

Bacteria Isolate	Total Number of Screened Isolates	Isolation Source	Strain ID
Aeromonas hydrophila	*39*	Water from Georgian Rivers, Lakes, Fish Farms	GW1-2; GW1-5; GW3-10; GW7-1; AW7-24; BW8-44; DW9-1; LW9-23; MW9-31; AW7-13; BW8-36; MW9-41;
		Diseased Fish from Georgian Farms	GF1-4; AF1-6; NF4-3; NF4-4; SF5-19; NF7-27; NF7-28; EF10-3; EF10-4; EF10-9; Iv24; N19; N37; N47; N48; N 53; AF2-5; GF3-1; AF4-7; SF5-18; Iv2; Iv3; Iv5
		Georgian Patients	a1; a2 a4; a5
Aeromonas caviae	*5*	Georgian Fish Farm Water	SW5-47; AW6-5; GW6-47; GW6-48; AW6-72
Aeromoans sobria	2	Georgian Fish Farm Water	GW1-3; AW2-1
*Aeromonas hydrophila**	*1*	River Water	CIP103770*
*Aeromonas salmonicida**	2	Fish	CIP 104001T*; CIP 103209T*

* *Aeromonas hydrophila* CIP103770, *Aeromonas salmonicida* CIP 104001T and *Aeromonas salmonicida* CIP 103209T from collection of Institute Pasteur

Table S2: Lytic activity of Bacteriophage AhMtk13a and AhMtk13b

N	Bacterial Isolate	AhMtk13a	AhMtk13b	Sours of isolation
1	*A. hydriphila GW1-2*	IP	R	Water
2	*A. hydriphila GF1-4*	CL	IP	Fish
3	*A. hydriphila GW1-5*	CL	R	Water
4	*A. hydriphila AF1-6*	CL	R	Fish
5	*A. hydriphila AF2-5*	R	R	Fish
6	*A. hydriphila GF3-1*	R	R	Fish
7	*A. hydriphila GW3-10*	CL	CL	Water
8	*A. hydriphila NF4-3*	CL	R	Fish
9	*A. hydriphila NF4-4*	CL	R	Fish
10	*A. hydriphila AF4-7*	R	R	Fish
11	*A. hydriphila SF5-18*	R	R	Fish
12	*A. hydriphila SF5-19*	CL	IP	Fish
13	*A. hydrophila GW7-1*	SCL	SCL	Water
14	*A. hydrophila AW7-13*	R	R	Water
15	*A. hydrophila AW7-24*	SCL	SCL	Water
16	*A. hydrophila NF7-27*	SCL	R	Fish
17	*A. hydrophila NF7-28*	CL	CL	Fish
18	*A. hydrophila BW8-36*	R	R	Water
19	*A. hydrophila BW8-44*	CL	SCL	Water
20	*A. hydrophila DW9-1*	SCL	R	Water
21	*A. hydrophila LW9-23*	SCL	R	Water
22	*A. hydrophila MW9-41*	R	SCL	Water
23	*A. hydrophila MW9-31*	OL	R	Water
24	*A. hydrophila EF10-3*	SCL	R	Fish
25	*A. hydrophila EF10-4*	SCL	R	Fish
26	*A. hydrophila EF10-9*	SCL	OL	Fish
27	*A. hydriphila Iv2*	R	R	Fish
28	*A. hydriphila Iv3*	R	R	Fish
29	*A. hydriphila Iv5*	R	R	Fish
30	*A. hydriphila Iv24*	CL	IP	Fish
31	*A. hydrophila N19*	SCL	R	Fish
32	*A. hydrophila N37*	SCL	SCL	Fish
33	*A. hydrophila N47*	SCL	R	Fish
34	*A. hydrophila N48*	SCL	R	Fish

35	*A. hydrophila N53*	SCL	SCL	Fish
36	*A. hydrophila a1(urine)*	SCL	SCL	Human
37	*A. hydrophila a2(nasal cavity)*	R	R	Human
38	*A. hydrophila a4 (sperm)*	SCL	R	Human
39	*A. hydrophila a5(urine)*	SCL	R	Human
40	*A. hydrophia CIP103770*	SCL	R	Water
41	*A. caviae SW5-47*	R	R	Fish
42	*A. caviae AW6-5*	R	R	Fish
43	*A. caviae GW6-47*	R	R	Fish
44	*A. caviae GW6-48*	R	R	Fish
45	*A. caviae AW6-72*	R	R	Fish
46	*A. sobria GW1-3*	R	R	Fish
47	*A. sobria AW2-1*	R	R	Fish
48	*A. salmonicida CIP104001T*	R	R	Fish
49	*A. salmonicida CIP 103209T*	R	R	Fish

CL, confluent lysis;

SCL, semi-confluent lysis;

OL, overgrown lysis;

IP, individual plaques;

R, Resistant;
